# Complementarity of two approaches based on the use of high-resolution mass spectrometry for the determination of multi-class antibiotics in water. Photodegradation studies and non-target screenings

**DOI:** 10.1007/s11356-022-22130-9

**Published:** 2022-08-04

**Authors:** Lua Vazquez, Maria Llompart, Thierry Dagnac

**Affiliations:** 1 CRETUS, Department of Analytical Chemistry, Nutrition and Food Science, Faculty of Chemistry, Universidade de Santiago de Compostela, 15782 Santiago de Compostela, Spain; 2 Agronomic and Agrarian Research Centre (AGACAL-CIAM), Unit of Organic Contaminants, Apartado 10, 15080 A Coruña, Spain

**Keywords:** Antibiotics, Solid-phase extraction, UHPLC-QToF-HRMS, Non-target analysis, Photodegradation, Photoproducts

## Abstract

The development of analytical methodologies to monitor different antibiotic families in water and the implementation of alternatives for their efficient elimination are a great challenge. The aim of this research was to develop a method based on solid-phase extraction followed by ultra-high performance liquid chromatography coupled to quadrupole time-of-flight mass spectrometry to analyse multi-class antibiotics, including macrolides, cephalosporins, fluoroquinolones, sulfonamides and diaminopyrimidines, in waters. Several parameters affecting the extraction such as the sample pH, type of sorbent and cartridge, elution volume and breakthrough volume were optimized. The method was validated in real samples, and matrix effect was assessed, demonstrating that the use of isotopically labelled surrogate compounds was mandatory to avoid standard addition calibration for each individual samples. Urban and hospital wastewater samples, as well as natural waters, were analysed, confirming the presence of 12 of the 14 target compounds at concentrations up to 3.5 µg L^−1^. Non-target analysis based on data-independent workflow was also performed, enabling the identification of 94 pollutants. Preliminary photodegradation experiments were also assessed, revealing the total removal of many target compounds after the first 5–10 min of UVC irradiation. In addition, 20 by-products formed after photolysis could be identified using a non-target approach.

**Supplementary Information:**

The online version contains supplementary material available at 10.1007/s11356-022-22130-9.

## Introduction

The presence and persistence of pharmaceuticals in water is growing, due to the increasing use of these substances by the population and in veterinary medicine (Čizmić et al. 2017; Kosma et al. 2020). Within these compounds, one of the most interesting groups is the antibiotics, which produce microbial resistance because of their irresponsible and irrational use, being mainly excreted as the active parent chemical. As in wastewater treatment plants (WWTPs), they are only partially removed; these substances are continuously entering into the environment (Berges et al. 2021; Carballa et al. 2004; Christou et al. 2017; Kosma et al. 2020) implying a significant risk to the aquatic ecosystem and human health. Alternative procedures such as photodegradation appear to be an option for the efficient removal of antibiotics and other emerging contaminants. Furthermore, it can be a cost-effective tertiary treatment compared to other ones such as advanced oxidation processes (AOPs), and, during UVC photolysis, no other reagents are needed for the degradation process, avoiding their further discharge into the aquatic environment (O_3_, H_2_O_2_, etc.). However, during photodegradation, by-products may be formed, so their identification is also of great importance (Batchu et al. 2014; Jia et al. 2021; Klementová et al. 2022; Sanchez-Prado et al. 2006; Wang and Lin, 2012). Although antibiotics are present as a mixture in the environment, the reported photodegradation studies were performed for only few antibiotics and at high concentrations (mg L^−1^) in comparison with the usual levels existing in real samples. Moreover, most studies only implied the evaluation of single analytes and substances of the same family.

The European Union Commission has established a Watch List under the Water Framework to monitor unregulated substances in the European waters, which can cause a significant risk to or via the aquatic environment (Loos et al. 2018). Currently, four antibiotics are included in the 3rd Watch List (amoxicillin, ciprofloxacin, sulfamethoxazole and trimethoprim) (Decision 2020/1161/EU, 2020). The European Union suggests using solid-phase extraction–ultra-high performance liquid chromatography tandem mass spectrometry (SPE-UHPLC-MS/MS) as analytical method for the monitoring of these substances in waters, although a specific method is not proposed (Decision 2015/495/EU 2015; Decision 2018/840/EU 2018; Decision 2020/1161/EU 2020). As reported in literature, SPE is the most employed technique regarding the sample preparation procedure (Čizmić et al. 2017; Gros et al. 2013; Martínez-Orgániz et al. 2021; Mokh et al. 2017; Tuc Dinh et al. 2011; Yuan et al. 2019; Zheng et al. 2022), and less studies apply other extraction techniques such as solid-phase microextraction (SPME) (Balakrishnan et al. 2006; McClure and Wong, 2007; Mitani and Kataoka, 2006), dispersive solid-phase extraction (d-SPE) (Herrera-Herrera et al. 2013a; Wang et al. 2019), liquid–liquid extraction (LLE) (Ashfaq et al. 2016) and dispersive liquid–liquid microextraction (DLLME) (Herrera-Herrera et al. 2013b). Regarding the analytical determination of antibiotics in wastewater, LC–MS/MS has been the most employed technique (Čizmić et al. 2017; Martínez-Orgániz et al. 2021; Mokh et al. 2017; Rossmann et al. 2014; Tong et al. 2009; Tuc Dinh et al. 2011; Yuan et al. 2019). Other detectors were also used, such as diode array detectors (DAD), Orbitrap mass spectrometry (Orbitrap MS), time-of-flight mass spectrometry (ToF–MS) and quadrupole time-of-flight mass spectrometry (QToF-MS) (Diaz et al. 2013; Kosma et al. 2020; Pedrouzo et al. 2008; Turiel et al. 2005; Tylová et al. 2013).

Among the SPE methods, different parameters have been studied to obtain the highest extraction efficiency and selectivity for different antibiotic families in water. The type of cartridge and the sample pH are some of the most evaluated factors (Čizmić et al. 2017; Gros et al. 2013; Rossmann et al. 2014; Tong et al. 2009). Nevertheless, most studies did not evaluate the elution volume, an important parameter to minimize the use of organic solvents and to achieve maximum extract enrichment, and the amount of sample. In addition, very few studies evaluate the accuracy in water samples of different characteristics, which could seriously compromise the validity of the results.

One of the aims of this work consisted of the optimization of the SPE-UHPLC-QToF methodology, to analyse and quantify antibiotic residues of different families in water; some of them included in the 3rd Watch List (ciprofloxacin, sulfamethoxazole and trimethoprim), as well as macrolides (azithromycin, clarithromycin, erythromycin and roxithromycin), which were removed from the 1st Watch List. Other antibiotics such as cephalosporins (cephalexin), fluoroquinolones (enrofloxacin, norfloxacin and ofloxacin) and sulfonamides (sulfadiazine, sulfadimidine and sulfamerazine) were targeted, as well. After being optimized, the method was extensively validated and applied to different water matrixes such as urban wastewater (effluent and influent), hospital wastewater and river and well water, collected in Galicia (NW Spain) and Porto (N Portugal). As second approach, a SWATH non-target analysis was carried out by means of UHPLC-QToF-HRMS for the identification of other antibiotics and other emerging pollutants. Preliminary photodegradation experiments and a tentative identification of the main photodegradation by-products were also conducted being, to our knowledge, the first study including the simultaneous photodegradation of such a high number (14) of multi-class antibiotics.

## Materials and methods

### Chemicals, materials and samples

Formic acid, methanol and ultrapure water MS grade were supplied by the Scharlab (Barcelona, Spain), and ammonium formate was from the Sigma-Aldrich Chemie GmbH (Steinheim, Germany). Ciprofloxacin (Cipro), enrofloxacin (Enro), norfloxacin (Nor), ofloxacin (Oflo), sulfadiazine (SDZ), sulfadimidine (SDMD), sulfamerazine (SMZ), sulfamethoxazole (SMX) and trimethoprim (TMP) were supplied by the Sigma-Aldrich Chemie GmbH (Steinheim, Germany); clarithromycin (Clar), erythromycin (Ery) and roxithromycin (Rox) by the Glentham Life Sciences Ltd. (Wiltshire, UK); and azithromycin (Azi) and cephalexin (Ceph) by the Apollo Scientific Ltd (Cheshire, UK). Clarithromycin-D3 (Clar-D3), norfloxacin-D5 (Nor-D5) and sulfamethoxazole-D4 (SMX-D4) were purchased from the LGC GmbH (Luckenwalde, Germany). Individual stock solutions of each compound (concentration about 1000 mg L^−1^) were prepared in methanol except for Cipro and Oflo, prepared in a water solution of HCl 0.4% and NaOH 1 M, respectively. Stock solutions were stored in glass vials and protected from light at − 20 °C. All solvents and reagents were of analytical grade. Target compounds, their acronyms, the CAS numbers, their LC retention times and the quantification and identification ions to ensure an unequivocal identification of the studied compounds are summarized in Table 1.

**Table 1:** Studied compounds. Acronym, CAS number, retention time, quantification and identification ions

Family	Antibiotic	Acronym	CAS number	Retention time (min)	Quantification ions	Identification ions
Macrolides	Azithromycin	Azi	83905–01-5	4.95	749.5158	591.4215, 158.1176, 116.107, 83.0491
	Clarithromycin	Clar	81103–11-9	8.14	748.4842	158.1176, 558.3637, 590.384, 83.0491
	Clarithromycin-D3	Clar-D3	959119–17-6	8.14	751.5030	590.3884, 158.1534
	Erythromycin	Ery	114–07-8	6.99	734.4685	116.107, 158.1176, 522.3425, 540.3531, 558.3637, 576.3742, 83.0491, 735.4719
	Roxithromycin	Rox	80214–83-1	8.33	837.5318	838. 5318, 158.1176, 679.4376, 116.107, 522.3273
Cephalosporins	Cephalexin	Ceph	15686–71-2	4.07	348.1013	106.0651, 158.027, 349.1041
Fluoroquinolones	Ciprofloxacin	Cipro	85721–33-1	4.24	332.1405	231.0564, 288.1507, 314.1299, 333.1435
	Enrofloxacin	Enro	93106–60-6	4.44	360.1718	245.1085, 316.182, 342.1612, 361.1749
	Norfloxacin	Nor	70458–96-7	4.19	320.1405	233.1085, 276.1507, 300.1343, 302.1299, 321.1435
	Norfloxacin-D5	Nor-D5	1015856–57-1	4.19	325.1719	307.1613, 326.1749
	Ofloxacin	Oflo	82419–36-1	4.16	362.1511	261.1034, 318.1612, 344.1405, 363.1541
Sulfonamides	Sulfadiazine	SDZ	68–35-9	3.83	251.0597	108.0444, 156.0114, 176.0124, 185.0822, 92.0495, 96.0556, 252.0622
	Sulfadimidine	SDMD	57–68-1	4.37	279.0910	108.0444, 124.0869, 156.0114, 186.0332, 204.0437, 92.0495, 92.0495
	Sulfamerazine	SMZ	127–79-7	4.12	265.0754	108.0444, 110.0713, 156.0114, 190.0281, 199.0978, 287.0573, 92.0495, 266.0779
	Sulfamethoxazole	SMX	723–46-6	4.49	254.0594	108.0444, 156.0114, 160.0869, 188.0818, 92.0495, 93.0573, 99.0553, 255.0620
	Sulfamethoxazole-D4	SMX-D4	1020719–86-1	4.49	258.0845	112.0695, 160.0365, 68.0574, 96.0746, 99.0553, 259.0871
Diaminopyrimidines	Trimethoprim	TMP	738–70-5	4.02	291.1452	123.0679, 230.1162, 245.1033, 261.0982, 275.1139, 292.148

Oasis HLB (60 mg, 3 mL), Oasis HLB (200 mg, 6 mL) and Oasis HLB Prime (150 mg, 3 mL) were from Waters (Milford, MA, USA). Strata-X (60 mg, 3 mL), Strata-X (200 mg, 6 mL) and Strata-X Pro (200 mg, 3 mL) were from Phenomenex (Torrance, CA, USA).

Different real water samples, including five effluents (EF) and an influent (INF) from urban WWTPs, two effluents from hospital wastewater (HW) and a river water (RW) located near a hospital as well as a well water (WW) from an agricultural-livestock area, were collected in Galicia (NW, Spain) and Porto (Portugal). Individual samples of 1 L were collected in amber glass bottles, and they were transported and stored at 4 °C in the dark. The samples were vacuum-filtered through 0.45-µm glass microfiber filters GF/A (70 mm diameter, Whatman, Kent, UK) before the extraction.

### Sample preparation and photodegradation experiments

The optimized procedure (see Fig. S1) was as follow: the Oasis HLB (60 mg, 3 mL) cartridges were conditioned with 2 mL of methanol followed by 2 mL of ultrapure water, and, then, 50 mL of sample acidified at pH 3 with HCl 2% (v/v) were loaded through the sorbent at a flow rate of 3 mL min^−1^, using vacuum with the SPE manifold (Visiprep™, Supelco, USA). Before drying the adsorbent under vacuum, cartridges were rinsed with 2 mL of ultrapure water. Once the cartridge was dried, the concentrated compounds were eluted with 1 mL of methanol. In the case of Oasis HLB (200 mg, 6 mL) cartridges, 200 mL of water and 5 mL of methanol as elution solvent were employed.

The study of the pH, the type of cartridges and breakthrough volume were carried out employing ultrapure water spiked at 2 µg L^−1^ with the target antibiotics. Regarding the elution volume assessment, the ultrapure water was fortified at 0.2 µg L^−1^, and the elution was achieved with two consecutive 1 mL fractions of methanol for Oasis HLB (60 mg, 3 mL) and 2.5 mL for Oasis HLB (200 mg, 6 mL). Matrix effect was also evaluated and isotopically labelled surrogates (Clar-D3, Nor-D5 and SMX-D4) were added to the samples (0.25 µg L^−1^).

In order to carry out the photodegradation experiments, 3 mL of ultrapure water containing the target compounds at 100 µg L^−1^ were placed in two quartz cuvettes that were located inside a lab-scale photoreactor equipped with two UVC lamp obtained from Philips, Holland (*λ* = 254 nm, 8 W and 11 W), at a distance of 9 cm from the lamps (see Fig. S2). Solutions were irradiated under UVC light for different times, and, afterwards, they were transferred to a glass vial and directly analysed by UHPLC-QToF. Dark tests (without UVC radiation) were also performed to ensure that no antibiotic degradation occurred in the absence of light.

To avoid false-positive findings, procedural blanks employing ultrapure water were carried out, both for SPE and photodegradation studies. In addition, solvent blanks were also daily analysed.

### Instrumentation

#### Target analysis and quantification

Target compounds were determined by ultra-high performance liquid chromatography (Elute UHPLC 1300) coupled to quadrupole time-of-flight mass spectrometry (QToF) (Compact-II Instrument, Bruker Daltonics, Bremen, Germany). The chromatographic separation was achieved with an Intensity Solo HPLC column C18-2 (100 mm × 2.1 mm, 1.8 µm; Bruker Daltonics, Bremen, Germany) and a precolumn, kept at 40 °C. The gradient elution is described in Table S1.

The mass spectrometer was operated in the electrospray ionization (ESI) in positive mode. Ion source conditions were as follow: capillary 4500 V, nebulizer 29 psi, drying gas 8 L min^−1^ and gas temperature 200 °C. The QToF mass spectrometer operated in broadband collision-induced dissociation (bbCID) acquisition mode that provided MS and MS/MS spectra at the same time. Spectra were recorded over the range m/z 30 − 1000 with a scan rate of 2 HZ. To obtain the MS spectra, the bbCID mode works with a low collision energy of 6 eV, and to acquire the MS/MS spectra, a high collision energy of 30 eV was applied. The system was operated by Compass HyStar software and O-TOF Control version 5.2. Quantitation and processing software was TASQ Version 2.1 (Build 201.2.4019).

#### Non-target analysis—photoproduct identification

Both the non-target analysis and the tentative identification of photoproducts were carried out using a UHPLC system (Shimadzu Nexera X2) consisting of two high-pressure pumps (LC-30AD) and a SIL-30AC autosampler. QToF-high-resolution mass spectrometry (QToF-HRMS) non-target detection was performed by a SCIEX (Ontario, Canada) TripleTOF®5600 + equipped with a DuoSprayTM ion source and an electrospray ionization (ESI) probe working in positive mode. The chromatographic separation was achieved with a Kinetex Biphenyl (50 × 2.1 mm, 2.6 µm) column from Phenomenex (Torrance, CA, USA), kept at 40 °C with a CTO-30 A column oven. The mobile phase consisted of water (A) and methanol (B), both buffered with 3 mM ammonium formate and both including formic acid (0.15%). The flow rate was kept to 0.20 mL min^−1^, employing an 8-min gradient elution profile (10% B to 100% B) reaching a total runtime of 15 min, and 10 µL of sample were injected in the analytical column.

As regards the HRMS conditions, the source temperature was set at 550 °C, the ion source gas at 42 (au, arbitrary units), the curtain gas at 32 (au) and the ion spray voltage floating at 5500 V. The HRMS workflow consisted of a TOF Full Scan, using 220 ms as accumulation time and 80 V as declustering potential in the ESI. Simultaneously, a data independent approach based on SWATH (sequential windowed acquisition of all theoretical fragment ion mass spectra) was performed. For that, a wide mass range (80–1200 Da) was divided in 30 mass windows with an accumulation time of 30 ms for each one. The resulting total scan time per cycle was 1.16 s. The declustering potential was set to 80 V, and the collision energy was 40 V with an energy spread of 20 V. The system was operated by Analyst®1.7.1 control software for data acquisition and by SCIEX OS 1.5.0.23389 for data treatment and library search.

## Results and discussion

### Evaluation of SPE procedure

One of the most crucial steps for the accurate determination of antibiotics in water is the extraction step. Parameters potentially affecting the SPE procedure were then optimized in order to achieve the best extraction efficiency. For this reason, the sample pH, cartridge type, sample and elution volume were evaluated in this study.

#### Influence of pH

**Fig. 1:**
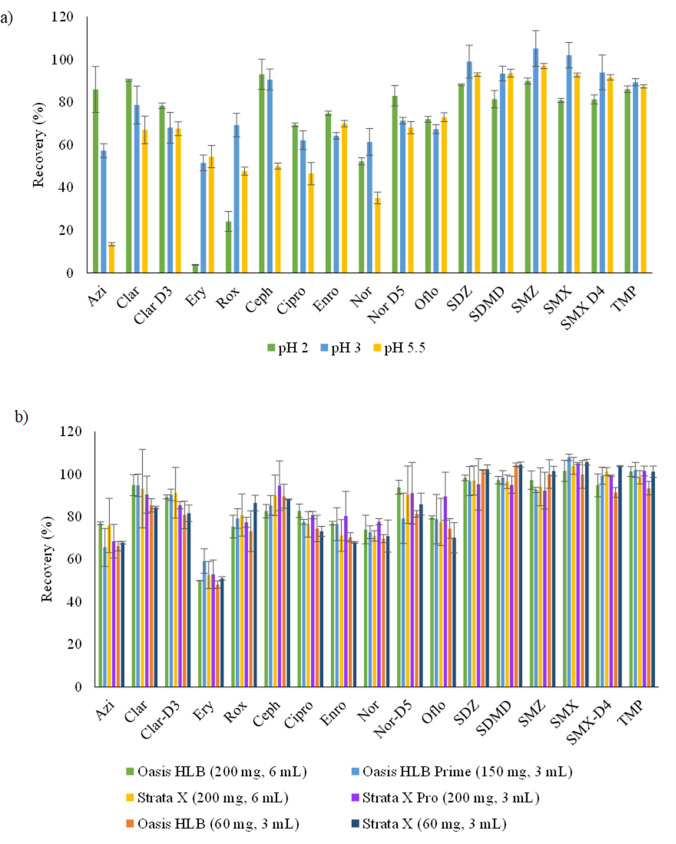
Recovery of the SPE procedure evaluating: **a** the influence of sample pH (2, 3 and 5.5) and **b** different types of cartridges

One of the most critical parameters affecting extraction efficiency of antibiotics in water is the sample pH. Although it has been included in other studies, in most cases the conclusions are not clear, especially for multi-class antibiotics. For this reason, the influence of the pH was evaluated (pH values 2, 3 and 5.5). Experimental conditions are described in the “Sample preparation and photodegradation experiments” section, and the obtained results are depicted in Fig. 1a. As it can be seen, the pH value was not significant for sulfonamide antibiotics, TMP and some fluoroquinolones (Enro and Oflo), which achieved high recovery values for all pH tested. On the other hand, although Azi, Clar and Cipro achieved higher recovery values at pH 2, compounds such as Ery, which according literature is converted into its main degradation product (Ery-H_2_O) at pH lower than 7 (Tuc Dinh et al. 2011), and Rox only reached values about 3 and 19%, respectively. By contrast, under pH 3, the recoveries of these two compounds were much higher, although for others, especially Azi, the efficiency decreased. When sample pH was 5.5, recoveries were lower for most of compounds, especially for Azi, Rox, Cipro and Nor. For this reason, and according to the pKa values and the reported results in other studies at higher pHs, the pH of the sample was not tested above 5.5 (Tlili et al. 2016). Thus, in order to reach a compromise for the simultaneous extraction of the target compounds, pH 3 was selected to perform further experiments since it allowed recovery values within acceptable ranges from 52 to 105% for all antibiotics. These results are in concordance with other studies in which acidic conditions with pH between 2.5 and 4 were chosen as the most efficient (Čizmić et al. 2017; Gros et al. 2013; Rossmann et al. 2014).

#### Type of cartridge

With the aim of attaining the highest extraction efficiency, four commercial polymeric SPE cartridges were tested: Oasis HLB (200 mg, 6 mL), Oasis HLB Prime (150 mg, 3 mL), Strata-X (200 mg, 6 mL) and Strata-X Pro (200 mg, 3 mL) (see experimental conditions in the “Sample preparation and photodegradation experiments” section). As can be seen in Fig. 1b (four first bars for each compound), no significant differences were observed among the tested cartridges and recoveries ranged between 48 and 108%.

On the other hand, as one of the recurrent objectives of sample preparation is to make the analytical methods as green and sustainable as possible, the use of lower amounts of sorbent, elution solvent and sample was tested employing Oasis HLB and Strata-X (60 mg, 3 mL). Figure 1b (two last bars for each compound) shows the recoveries obtained for both cartridges compared with those obtained with the previous four ones. The results were similar for all compounds. Therefore, these last two cartridges were selected as the best choice since they enable a scale down method with less consumption of sorbent (60 mg instead of 200 mg) and organic solvent (1 mL instead of 5 mL) and a higher enrichment factor.

The efficiency of commercially packed cartridges and manual packed cartridges prepared in the lab was compared using the same materials. Manual packed cartridges were prepared with 60 mg of sorbent, in a 2 mL polypropylene cartridge with a cellulose filter at the top and bottom. For all target antibiotics, recovery values were lower, with fluoroquinolone antibiotic percentages dropping up to 20 times (Fig. S3). Thus, the commercial packed cartridges were selected to perform the SPE of antibiotics. For the following experiments, it has been decided to work with Oasis HLB (60 mg, 3 mL) since several studies reported this type of cartridge as the best option to carry out the extraction of antibiotics in different water samples (Čizmić et al. 2017; Diaz et al. 2013; Gros et al. 2013). In addition, the use of Oasis HLB with more sorbent capacity (200 mg, 6 mL) was optimized and validated since it remains one of the preferred cartridges for the determination of these compounds (Mokh et al. 2017; Tlili et al. 2016).

#### Elution volume

In order to achieve the optimum enrichment factor of the target compounds and, taking into account that in most studies the elution is carried out with a higher volume of solvent (Čizmić et al. 2017; Gros et al. 2013), the elution volume influence was assessed collecting two consecutive and separate fractions of the SPE elute (see the “Sample preparation and photodegradation experiments” section). Recovery values for Oasis HLB (60 mg, 3 mL) depicted in Fig. S4a demonstrate that 1 mL of methanol was enough to elute the target compounds. In this case, recoveries in the second fraction were lower than 2%. Hence, the use of 1 mL of methanol was selected for Oasis HLB (60 mg, 3 mL), since it allowed a high enrichment factor and a lower consumption of organic solvent.

As regards Oasis HLB (200 mg, 6 mL), only three compounds were detected in the second fraction, as it is shown in Fig. S4b. However, as Clar recovery in the second 2.5 mL fraction was not negligible (7%), 5 mL of solvent was selected as elution volume in this particular case.

#### Breakthrough volume

Another important SPE parameter is the breakthrough volume that determines the maximum sample volume that can be loaded on the sorbent without analyte loss. Under the optimum conditions using Oasis HLB (60 mg, 3 mL) (see the “Sample preparation and photodegradation experiments” section), different water sample volumes were evaluated: 10, 25, 50, 100 and 200 mL. As shown in Fig. S5, no breakthrough was observed for most of the target antibiotics. However, recovery values for Ery and Ceph clearly decreased when the sample volume was greater than 50 mL, and recoveries of Rox, Enro and TMP started to decrease, as well. Therefore, 50 mL was selected as the optimum sample volume to perform the SPE procedure followed by the elution with 1 mL of MeOH, which implies an enrichment factor of 50 in case of quantitative recoveries. Other studies reported higher sample volumes, although they did not assess the breakthrough volume (Čizmić et al. 2017; Diaz et al. 2013). In addition, the use of a 50 mL volume reduces the sample preparation time. On the other hand, if the expected antibiotic concentrations are high (e.g. in hospital wastewaters), and the available sample volume is limited, lower volumes can be used for the extraction procedure.

### Method performance parameters

Calibration study was performed employing standard solutions prepared in methanol, containing the target compounds at different levels, covering a concentration range from 0.05 to 2000 µg L^−1^ (see specific ranges for each compound in Table 2) with fourteen levels and three replicates per level. The method showed a direct proportional relationship between the concentrations of each analyte and the chromatographic responses with coefficients of determination (*R*^2^) higher than 0.9922 in all cases. The calibration plots for each target compound and their spectra at the low concentration levels are depicted in Fig. S6 and Fig. S7, respectively. The instrumental precision was evaluated within a day (*n* = 4) and among days (*n* = 6) at different concentration levels. RSD values for 50 µg L^−1^ are shown in Table 2. As can be seen, RSD values were for most compounds lower than 8 and 10% for intra- and inter-day precision, respectively.

**Table 2:** Method performance. Linearity, precision, mean recoveries in ultrapure water, LODs and LOQs

Compound	Linearity		Recoveries in ultrapure water (%)				Mean recoveries in real samples (%)	Precision		LODs (ng L^−1^)	LOQs (ng L^−1^)
	Range (µg L^−1^)	*R*^2^	0.2 µg L^−1^		2 µg L^−1^			RSD (%)			
			60 mg	200 mg	60 mg	200 mg		Intra-day (*n* = 4)	Inter-day (*n* = 6)		
Azi	0.1–1000	0.9961	64.3 ± 6.2	60.4 ± 2.3	66.1 ± 2.2	76.85 ± 0.67	104 ± 15	7.8	15	0.76	2.5
Clar	0.5–2000	0.9986	78.7 ± 4.5	77.3 ± 1.4	85.3 ± 3.1	94.8 ± 4.9	102.5 ± 5.6	2.5	15	2.8	9.3
Clar-D3*			*73.5* ± *3.3*	*75.5* ± *4.3*	*75.1* ± *1.7*	*89.26* ± *0.93*		*1.1*	*11*	*1.2*	*4.1*
Ery	0.2–2000	0.9959	41.8 ± 4.6	39.2 ± 2.9	48.1 ± 1.7	49.92 ± 0.10	85.8 ± 7.0	2.1	7.6	3.0	10
Rox	0.2–2000	0.9993	61.5 ± 2.9	60.59 ± 0.28	73.2 ± 9.6	75.3 ± 5.3	100.9 ± 3.6	1.3	11	1.3	4.3
Ceph	5–2000	0.9962	86.43 ± 0.51	93.58 ± 0.92	89.5 ± 5.6	82.6 ± 3.2	78.2 ± 5.4	10	7.6	28	92
Cipro	0.2–2000	0.9948	78.4 ± 7.0	81.2 ± 4.8	78.8 ± 6.2	82.8 ± 3.2	89 ± 17	13	9.6	1.2	3.8
Enro	0.2–2000	0.9922	78.8 ± 6.0	76.1 ± 5.2	70.3 ± 2.2	76.87 ± 0.86	109 ± 16	7.3	7.5	1.2	3.7
Nor	1–2000	0.9926	73.0 ± 1.4	76.6 ± 4.6	69.7 ± 1.7	73.9 ± 6.7	77 ± 15	8.7	7.4	7.0	23
Nor-D5*			*74.1* ± *7.0*	*82.7* ± *3.9*	*81.3* ± *1.5*	*93.8* ± *3.3*		*4.9*	*9.5*	*13*	*42*
Oflo	0.2–2000	0.9925	79.3 ± 4.4	73.81 ± 0.30	74.4 ± 4.6	79.7 ± 0.7	102 ± 11	6.1	12	1.3	4.4
SDZ	0.05–1000	0.9966	98.3 ± 5.4	96.1 ± 4.3	101.9 ± 1.1	98.2 ± 1.2	101.0 ± 9.0	2.9	8.0	0.37	1.2
SDMD	0.05–500	0.993	90.5 ± 1.8	93.96 ± 0.13	104.4 ± 1.0	97.2 ± 1.7	94 ± 12	2.2	4.4	0.33	1.1
SMZ	0.1–1000	0.9955	89.8 ± 2.8	97.4 ± 4.3	100.0 ± 6.7	97.2 ± 4.3	96.8 ± 9.8	1.2	8.7	0.70	2.3
SMX	0.05–1000	0.9923	85.31 ± 0.93	96.0 ± 1.0	99.8 ± 6.6	101.5 ± 5.0	96.0 ± 5.8	3.0	11	0.40	1.3
SMX-D4*			*95.9* ± *4.3*	*93.6* ± *1.6*	*91.3* ± *2.3*	*94.7* ± *5.3*		*3.4*	*9.0*	*1.7*	*5.6*
TMP	0.05–500	0.9927	91.4 ± 1.7	98.2 ± 3.4	93.4 ± 3.1	101.2 ± 2.3	93 ± 19	2.7	9.9	0.31	1.0

^*^Isotopically labelled surrogates were added at 0.25 µg L^**−**1^

Recovery studies were carried out in ultrapure water at two fortification levels: 0.2 and 2 µg L^−1^ including all the target antibiotics. In all cases, fortifications were performed by triplicate employing two different SPE cartridges, Oasis HLB (60 mg, 3 mL) and Oasis HLB (200 mg, 6 mL), and were calculated by comparing the obtained chromatographic responses (area counts) with those obtained with standards prepared in methanol. As can be seen in Table 2, recoveries ranged between 60 and 104% with RSD values lower than 10% for all target compounds, excluding erythromycin, giving recoveries about 40–50%. As an example, the spectra and chromatogram for Nor and Nor-D5 are depicted in Fig. S8.

The LODs and LOQs of the method were calculated as the compound concentration giving a signal-to-noise ratio of three (S/N = 3) and ten (S/N = 10), respectively, since the target antibiotics were not detected in the whole method blanks. Oasis HLB (60 mg, 3 mL) and ultrapure water spiked at the lowest concentration level were employed to calculate the limits LODs and LOQs shown in Table 2, obtaining satisfactory results.

#### Antibiotic recoveries in waters of different origin

**Fig. 2:**
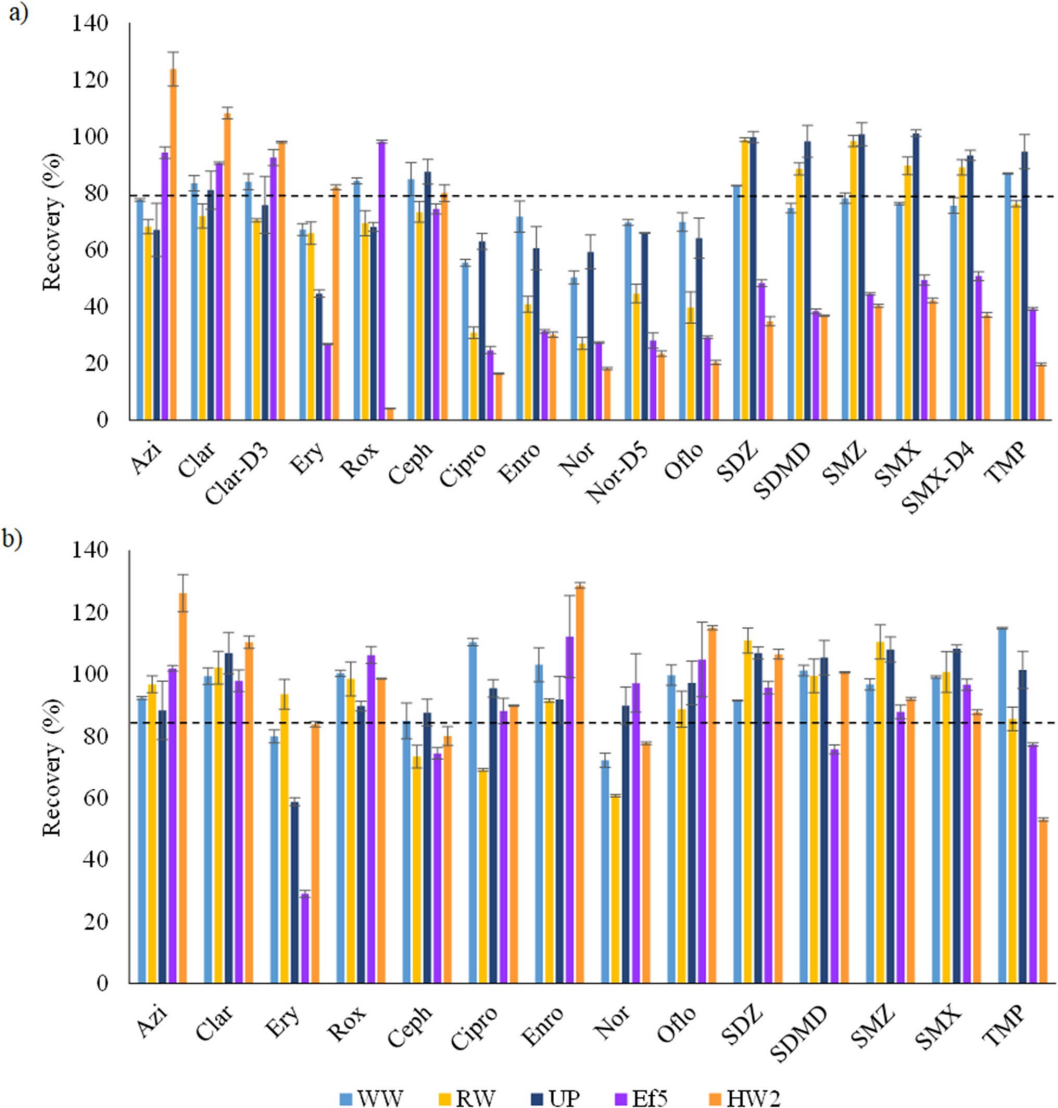
Antibiotic recoveries obtained for real water matrixes fortified at 1 µg L ^−1^ for well (WW), river (RW) and ultrapure (UP) water and at 5 µg L ^−1^ for effluents from urban (EF5) and hospital (HW2) wastewater treatment plants: **a** without considering isotopically labelled surrogates Clar-D3 (Azi, Clar, Ery, Rox), Nor-D5 (Cipro, Enro, Nor, Oflo) and SMX-D4 (SDZ, SDMD, SMZ, SMX, TMP) and **b** considering isotopically labelled surrogates

In order to assess the influence of the water matrix on the extraction process, recovery studies were performed in four real water matrixes of different origin at two concentration levels: 1 µg L ^−1^ in well (WW) and river (RW) and 5 µg L ^−1^ in effluents from urban (EF5) and hospital (HW2) wastewater treatment plants. In those cases, in which the compounds were initially detected in the samples, their responses were subtracted. Recovery values for ultrapure and the other water sample matrices are summarized in Fig. 2a. As it is observed, recovery values ranged between 40 and 90% for most compounds, although especially for the most complex matrixes (urban and hospital wastewater), recoveries decrease for sulfonamides and TMP, as well as for fluoroquinolone antibiotics. Thus, these differences observed between the recoveries obtained in different real water samples clearly show that the type of water matrix influences the extraction yield.

In an attempt to find out whether the low values were due to a low extraction efficiency or to matrix effects into the MS ionization source, some additional experiments were performed. SPE was applied to ultrapure water, and the obtained methanol extracts were spiked at two different levels and compared with standard solutions. According to the results (Fig. S9a), no matrix effect occurred into the ionization source since responses were comparable between the fortified extracts and the standard solutions. In addition, recovery values between the spiked extracts and the obtained extracts after SPE using fortified ultrapure water were compared (Fig. S9b). Recovery values for extracts spiked after SPE were 40% higher for Ery, demonstrating that the poor retention capacity of the sorbent was responsible of the low recoveries.

Consequently, isotopically labelled surrogate antibiotics have to be used to mitigate matrix effects. Three isotopically labelled compounds were selected according to their similarity with the compounds under study. In this way, Clar-D3 was used to correct matrix effects for all macrolide antibiotics, Nor-D5 for fluoroquinolones and SMX-D4 for sulfonamides and TMP. Recovery values for Ceph were not corrected since they were higher than 73% in all matrixes. As shown in Fig. 2b, the recoveries calculated using the isotopically labelled surrogates were quantitative for most compounds in the different water matrixes, achieving values above or close to 80% in most cases even in complex matrices such as hospital and urban wastewater. In Table 2, the mean recovery values in real samples are included (individual values for each real sample can be seen in Fig. 2b).

### Occurrence of antibiotics in water samples of different origin.

**Fig. 3:**
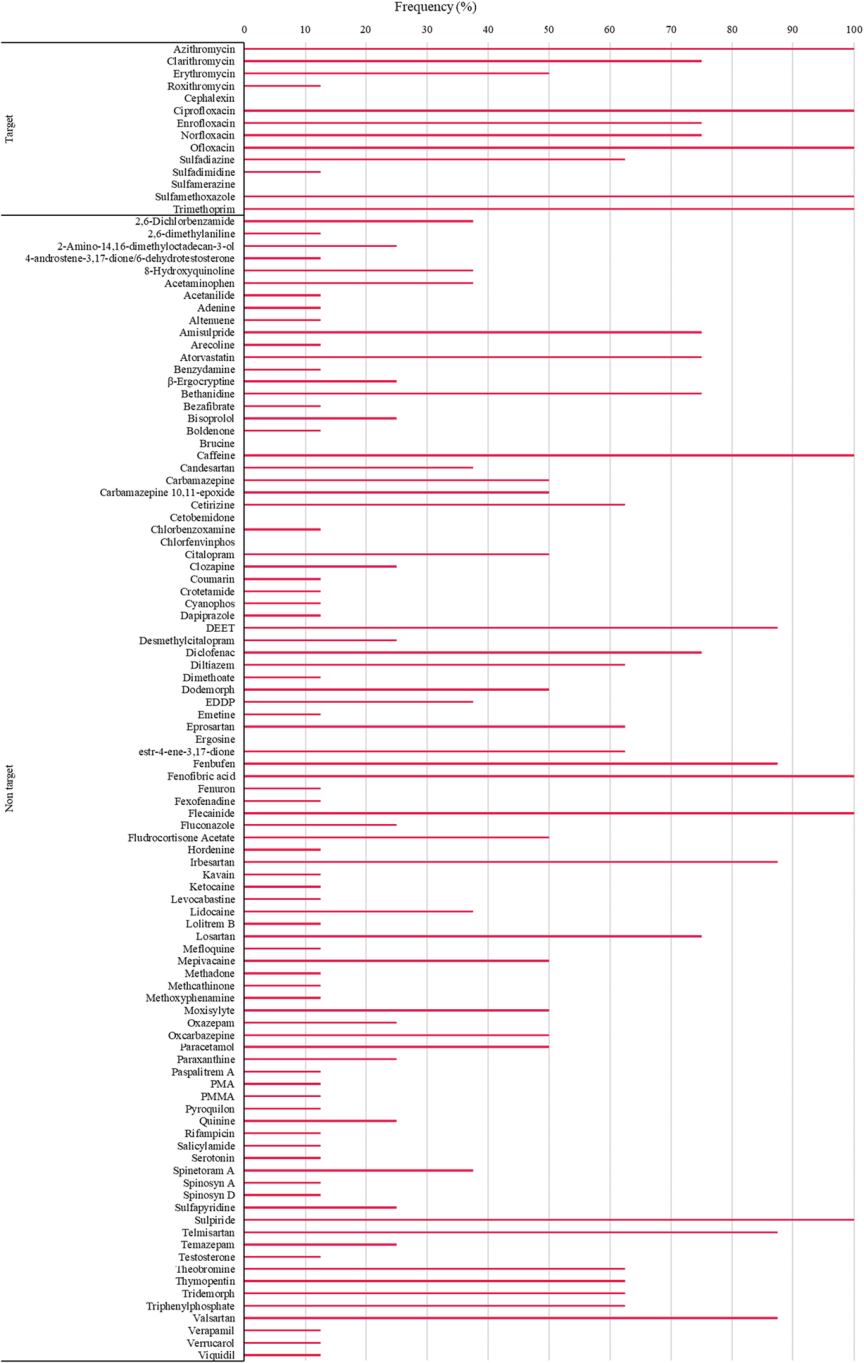
Detection frequency of target and non-target compounds in urban and hospital wastewaters

The presence of the studied macrolides, cephalosporins, fluoroquinolones, sulfonamides and diaminopyrimidines in real samples of different origin including urban wastewaters (influent, INF and effluents, EF), hospital wastewater (HW), river (RW) and well water (WW) was investigated. Results are summarized in Table 3, and the detection frequency of the compounds in the urban and hospital wastewater samples is depicted in Fig. 3.

**Table 3:** Concentrations (µg L^−1^) of the target antibiotics in different water samples. *EF* urban effluent, *INF* urban influent, *HW* hospital effluent, *WW* well water, *RW* river water

Compounds	EF1	EF2	EF3	EF4	EF5	INF1	HW1	HW2	WW	RW
Azi	1.3	0.15	0.49	0.67	0.81	0.056	3.4	1.9	0.0033	0.0077
Clar	0.14		0.24	0.11	0.19	0.10		0.034		
Ery	0.11				0.064	0.072		0.063		
Rox					0.043					
Cipro	0.15	0.10	0.22	0.11	0.14	0.080	0.66	0.18	0.23	
Enro	0.15	0.12	0.12	0.14	0.17	0.24			0.10	
Nor	0.22	0.15	0.27	0.28		0.83		0.21	0.072	
Oflo	0.88	0.074	0.19	0.62	0.18	0.43	3.5	2.5	0.19	
SDZ	0.010		0.010	0.010		0.095	0.35			
SDMD				0.0055						
SMX	0.12	0.029	0.023	0.091	0.15	0.087	0.10	0.073		
TMP	0.077	0.018	0.025	0.048	0.074	0.042	0.20	0.13	0.0016	0.019
∑antibiotics	3.2	0.64	1.6	2.1	1.8	2.0	8.2	5.1	0.60	0.027

All target compounds were found in the samples except Ceph and SMZ. Azi and TMP were present in all the analysed samples, followed by Cipro and Oflo detected in 9 out of the 10 samples and Enro, Nor and SMX in 8 samples. Clar, SDZ and ERY were only present in wastewaters, at 75, 63 and 50% detection frequencies, respectively (see Fig. 3). On the other hand, ROX and SDMD were only detected in one sample (EF5 and EF4, respectively). Regarding the number of compounds per sample in two urban effluents (EF1, EF4) and in INF, up to 10 compounds of the 14 targets were detected. It is important to note that EF4 and INF were collected at a WWTP serving around 400,000 habitants, which can influence the presence of a high number of the target compounds. The other samples contained between 2 and 9 target compounds. Although the hospital effluents (HW1 and HW2) were not the samples with the highest number of detected compounds, they contained the highest concentrations in terms of sum of the target antibiotics (8.2 and 5.1 µg L^−1^, respectively). Additionally, both hospital effluents also achieved the highest levels for some individual compounds, such as Azi and Oflo with concentrations up to 3.5 µg L^−1^. The concentrations found by other authors in real water samples are included in Table S2. The high concentrations for Oflo in hospital wastewater were in line with those reported by Gros et al. (2013) and the concentrations of the individual compounds in the effluent samples, which ranged between 0.0055 and 1.3 µg L^−1^, were also similar to those obtained in other studies (Gros et al. 2013; Rossmann et al. 2014; Tylová et al. 2013). On the other hand, as it could be expected, fewer antibiotics were generally detected in river and well water and at lower concentrations (0.027 and 0.61 µg L^−1^ for the sum, respectively) than in wastewaters. The presence of 6 antibiotics in the well water sample may be due to its location in an agricultural-livestock area. Among the detected antibiotics, TMP is classified as prudent use drugs by the Antimicrobial Advice Ad Hoc Expert Group (AMEG) and the other four (Cipro, Enro, Nor and Oflo) as antibiotics for use with limitation in cattle (European Medicines Agency 2019).

Therefore, these results clearly confirm the entrance of antibiotics into the environment, implying a significant risk to the aquatic environment and human health.

#### Screening studies by a non-target SWATH approach

Unknown compounds were identified employing a data retrospective approach and spectral libraries containing more than 2,000 high-resolution mass spectra. The SWATH workflow uses an identification protocol based on the exact masses of the unknown eluted compounds taking into account additional criteria for the confirmation such as the isotope profile and library score calculated with high-resolution MS/MS spectra. The combination of the three above-mentioned criteria provided a final combined score. All results showing a combined score below 50 were excluded from the final list of the tentatively identified compounds. The non-targeted compounds detected in the real samples and the related identification parameters are included in Table S3. The detection frequency of the non-target substances in the wastewater samples (urban and hospital) are summarized in Fig. 3.

Up to 94 compounds including antibiotics, anticonvulsants, analgesic, anti-inflammatory, β-blockers, stimulants and metabolites or pesticides among others were found in the analysed samples. A high number of these compounds were found in the analysed samples (Table S3), except in the well and river waters (6 and 10 compounds, respectively). As can be seen in Fig. 3, the most frequently detected compounds were the stimulant caffeine, the lipid-lowering agent fenofibric acid, the antiarrhythmic flecainide and the antipsychotic sulpiride, detected in the 100% of the wastewater samples, followed by the angiotensin receptor blockers irbesartan, telmisartan and valsartan; the insect repellent DEET; and the non-steroidal anti-inflammatory fenbufen identified in 88% of them.

### Photodegradation experiments

As it has been demonstrated, antibiotics can easily reach the aquatic environment; therefore, the use and improvement of water decontamination procedures is of great interest to protect the environment. Thus, a preliminary study dealing with the simultaneous photodegradation of the 14 target multi-class antibiotics was carried out. The water solutions (100 µg L^−1^) were irradiated under UVC light in a lab-scale photoreactor during different times: 5, 10, 20, 30, 40 and 60 min. The detailed experimental conditions are summarized in the “Sample preparation and photodegradation experiments” section.

**Fig. 4:**
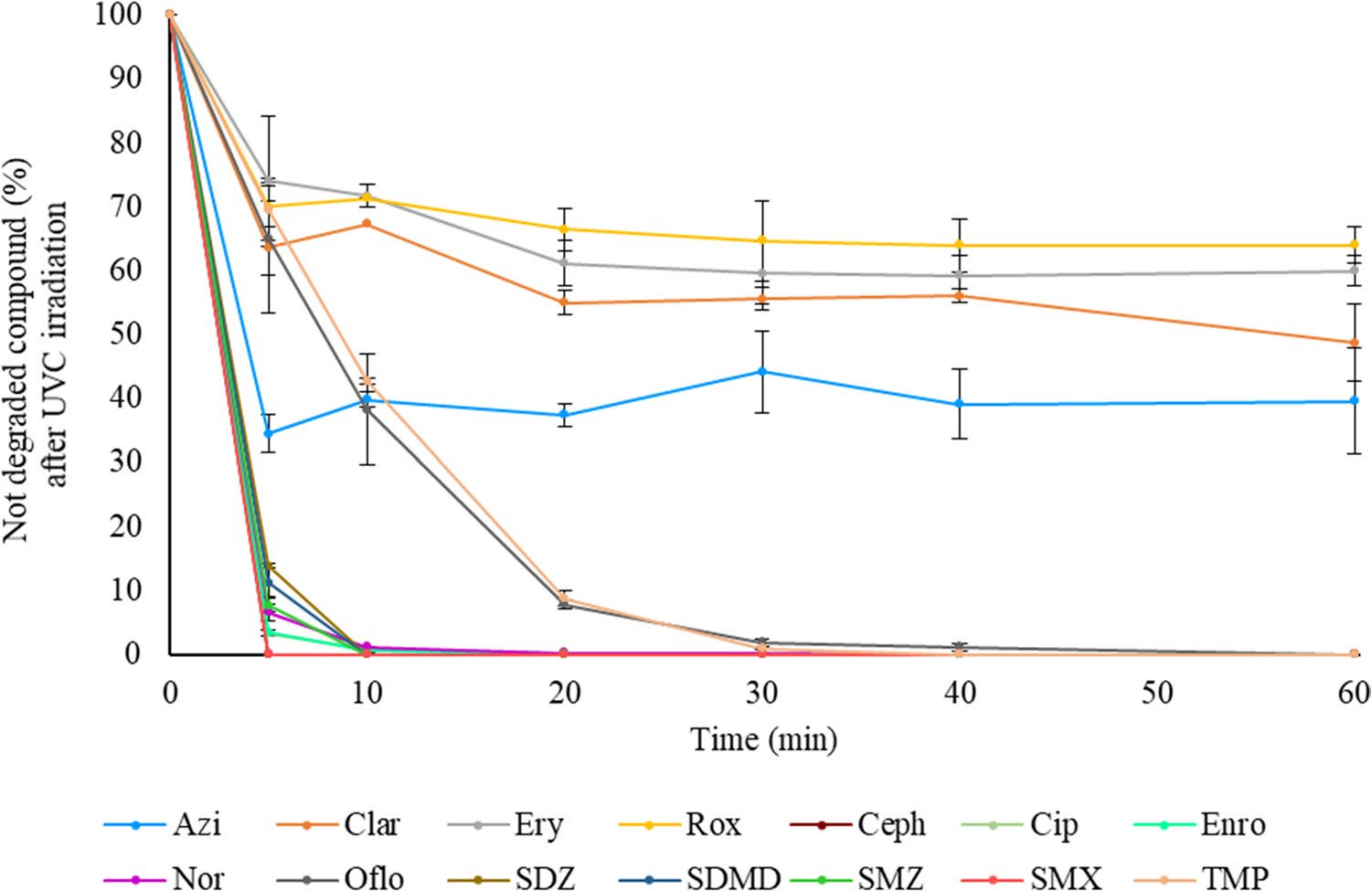
Degradation kinetics observed for the 14 studied antibiotics under UVC irradiation. Initial concentration: 100 µg L^−1^

The degradation kinetics for the antibiotics are depicted in Fig. 4. As can be seen, Ceph and SMX achieved complete degradation before the first 5 min. The other sulfonamide antibiotics and Enro were completely degraded after 5 min and Cipro and Nor after 10 min. TMP and Oflo reached a complete degradation, after 30 and 40 min, respectively. On the other hand, the macrolide degradation was much slower, reaching maximum degradation of 60% within the first 5–20 min and remaining at the same percentage for all the time (60 min). Results were in concordance with those reported by Batchu et al. (2014) for UVC photolysis, also demonstrating the slow degradation of macrolide antibiotics. On the other hand, other authors described protocols requiring longer times, even several hours, for the removal of some of the target compounds by photodegradation, employing different reactor types (Guo et al. 2013; Haddad and Kümmerer, 2014; Sirtori et al. 2010; Trovó et al. 2009).

Therefore, photodegradation seems to be a plausible option for the removal of most of the target antibiotics in water, and further studies including advanced oxidation processes could lead to efficient and novel procedures (Anjali and Shanthakumar, 2019). Nevertheless, it is necessary to assess the possible generation of photoproducts as well as their stability.

#### Tentative identification of photoproducts by QToF-(SWATH)-HRMS

In addition to UHPLC for separation purposes, the triple TOF mass spectrometer was employed, implementing a data independent workflow (SWATH) to identify and characterize the highest number of photodegradation by-products of the targeted antibiotics. This non-targeted retrospective search of the maximum number of unexpected ions present in the samples enables performing fragmentation of all precursor ions entering the mass spectrometer in 20–30 m/z isolation windows.

A comprehensive bibliographic research dealing with degradation studies conducted with the targeted antibiotics in different aqueous media and taking into account different types of photodegradation processes (solar radiation simulation or photocatalytic degradation) was carried out (Guo et al. 2013; Haddad and Kümmerer 2014; Jia et al. 2021; Sirtori et al. 2010; Song et al. 2017; Sturini et al. 2012; Trovó et al. 2009; Wang et al. 2018; Wang and Lin 2012; Zhang et al. 2019). It is important to underline that all these investigations only considered a few antibiotics and most of them dealt with only individual compounds or individual families. Therefore, it appears very necessary to develop multi-antibiotic determination methods since these substances are present simultaneously in natural waters. Hence, based on this quite exhaustive literature searching regarding the degradation of the 14 targeted precursor antibiotics, about 182 likely by-products could be identified.

As first approach, the by-products identification was achieved after importing to SCIEX-OS software—Analytics a list (actually, chemical formula—m/z precursor ions were imported) of the 182 suspected targeted by-products and metabolites. Twenty out of the 182 proposed by-products could be identified based on their exact mass and on the main fragments in their high-resolution MS/MS spectra. The molecular formula, precursor ion (m/z), mass error, structure and the bibliographic references of the identified photoproducts are summarized in Table 4.

**Table 4:**
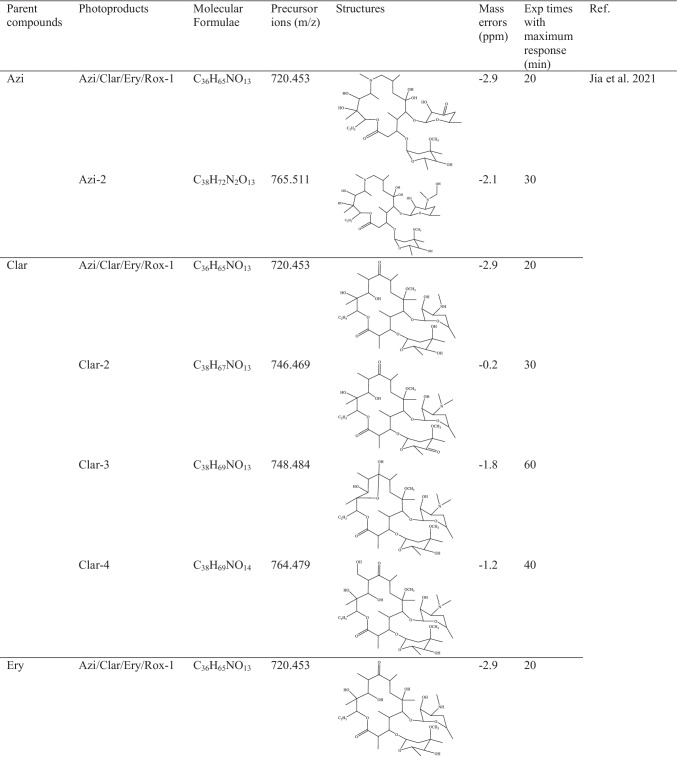

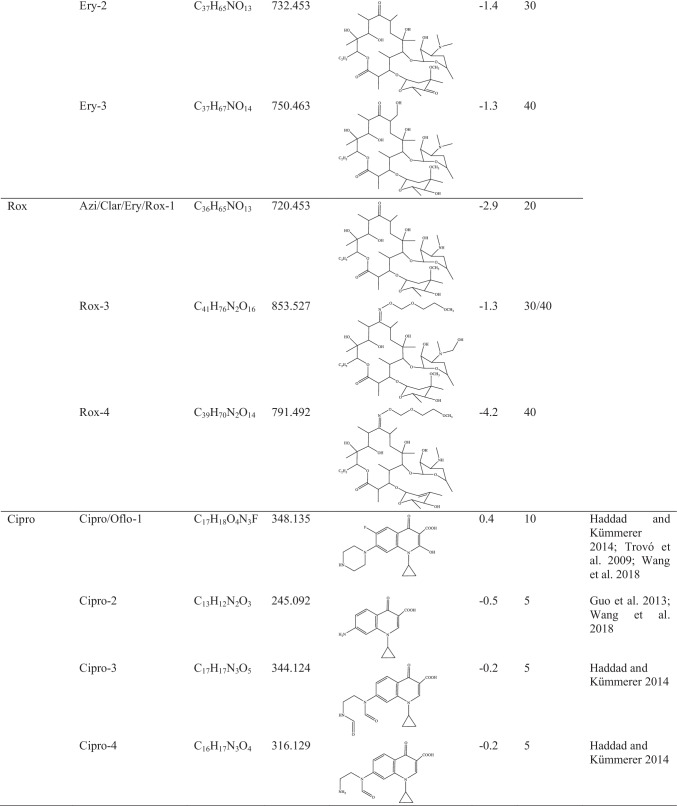
Data for the photodegradation products: parent compounds, photoproducts, molecular formulae, precursor ions, structures, mass errors, exposure times with maximum response and bibliographic references

Most of the identified by-products were detected at the maximum intensity within the first 5 and 10 min, except the transformation products from macrolides.

Among the 20 detected by-products, 7 of them were transformation products of fluoroquinolones, all of them reaching the maximum intensity at the experimental times of 5 and 10 min. Cipro was the fluoroquinolone from which the highest number of degradation products could be identified (4), which arose from the breakdown of the piperazine ring, hydroxylation, and substitution of fluorine and defluorination. The photoproduct obtained from Oflo has the same molecular formula as one stemming from Cipro, hence the same exact mass (C_17_H_18_O_4_N_3_F, m/z 341.135).

On the other hand, up to 12 transformation products of the four targeted macrolides could also been detected and identified. The chemical formulas were imported from a recent study conducted by Jia et al. (2021), in which the transformation products of these four compounds were comprehensively evaluated. The identified by-products appeared with the highest intensity after 20 and 40 min of irradiation, while one of them (Clar-3) reached the highest intensity at the maximum study time (60 min). Most of the degradation reactions involved demethylation, hydroxylation or combination of both. Rox can also degrade into Ery as first stage, the latter giving rise to a final by-product with m/z 720.4528 as exact mass (C_36_H_65_NO_13_). The main fragment observed in the MS/MS spectrum at m/z 562.3533 resulted from the loss of cladinose moiety. In addition, one by-product for each macrolide antibiotic could be identified with this chemical formula and exact mass (see Table 4). Therefore, it was not possible to differentiate them by HRMS without comparing their responses with those of their related standards.

The last characterized transformation product was identified as a by-product of SMZ. It is formed by cleavage of the S–N bond, and its maximum intensity was detected after 20 min of irradiation.

The highest responses were reached for two degradation products of Clar and one of Ery, with m/z 748.4842 (C_38_H_69_NO_13_), m/z 764.4791 (C_38_H_69_NO_14_) and m/z 750.4634 (C_37_H_67_NO_14_), as exact masses, respectively. The MS/MS spectra of these three degradations products also showed a main fragment resulting from the loss of cladinose moiety. As examples, the chromatographic peaks, isotopic profiles and MS/MS spectra of 4 of the identified by-products are shown in Fig. S10.

As second approach, a full non-targeted search was conducted using the same SWATH acquired data but without entering any candidate precursor ion of likely by-products. The not irradiated “t = 0” water sample was taken as reference sample. In this case, additional criteria besides exact mass of precursor ion were employed, such as mass accuracy of fragments, isotope score and formula finder score. High-resolution MS/MS spectra were also available in some cases.

About 30 precursor ions were identified in the different water samples in which photodegradation products were studied (irradiation times 5, 10 or 20 min). Among these 30 precursors, 7 were already present in the list of the 20 by-products previously identified (Table 4), thereby confirming the presence of these 7 degradation products. These precursor ions were m/z 245.093 (Cipro-2), 316.133 (Cipro-4), 348.135 (Cipro/Oflo-1), 732.451 (Ery-2), 750.463 (Ery-3), 764.478 (Clar-4) and 765.510 (Azi-2).

For the remaining 23 precursors, although the exact mass was established, it was not possible to identify the chemical structure or the compound from which they were formed. With this full non-targeted approach, it would be necessary to carry out studies for each individual compound. Nevertheless, the results showed that this tool could offer an alternative to identify transformation products.

## Conclusions

A solid-phase extraction methodology followed by ultra-high performance resolution liquid chromatography-quadrupole time-of-flight mass spectrometry (UHPLC-QToF-MS) was optimized for the simultaneous analysis of macrolides, cephalosporins, fluoroquinolones, sulfonamides and diaminopyrimidines in water samples. The optimum conditions implied the use of Oasis HLB (60 mg, 3 mL) as solid-phase extraction, 50 mL of sample acidified to pH 3 and 1 mL of methanol as elution solvent. The methodology was successfully validated, and matrix effects were assessed demonstrating that the use of isotopically labelled surrogates was necessary. Finally, the validated method was applied to different water matrixes including urban (effluent and influent) and hospital wastewater, well water and river water, putting in evidence the presence of all target antibiotics in these real samples. In addition, a non-target approach based on a SWATH workflow was performed, demonstrating the presence of up to 94 compounds with a wide variety of substances including pesticides, drugs, and pharmaceuticals in all samples. The UVC photodegradation kinetics showed the total removal of many target compounds after less than 30 min of irradiation, and twenty photodegradation by-products could be identified by means of the same SWATH workflow. Therefore, photodegradation appears as a good alternative to simultaneously remove the target antibiotics in ultrapure water, although studies in other water matrixes are also needed. However, for some compounds, especially macrolide antibiotics, which remained partially no degraded, advanced oxidation processes (AOPs) are deemed necessary to effectively remove these compounds and prevent them from reaching aquatic compartments.

## Supplementary Information

Below is the link to the electronic supplementary material.

Supplementary file1 (DOCX 2316 KB)

## Data Availability

Data are available within the present article and Supplementary Materials.
