# Supplementary material for: Complementarity of two approaches based on the use of high-resolution mass spectrometry for the determination of multi-class antibiotics in water. Photodegradation studies and non-target screenings
Source: Environ Sci Pollut Res Int. 2022 Aug 4;30(1):1871–88. doi: 10.1007/s11356-022-22130-9 (PMC9813094; doi:10.1007/s11356-022-22130-9)
Supplement: Supplementary file 1 — Supplementary file1 (DOCX 2316 KB) [file 11356_2022_22130_MOESM1_ESM.docx]

**Complementarity of two approaches based on the use of High-Resolution Mass Spectrometry for the determination of multi-class antibiotics in water. Photodegradation studies and non-targeted screenings**

Lua Vazquez^1^, Maria Llompart^1*^, Thierry Dagnac^2^

^1^CRETUS, Department of Analytical Chemistry, Nutrition and Food Science, Faculty of Chemistry, University of Santiago de Compostela, E-15782, Santiago de Compostela, Spain.

^2^Agronomic and Agrarian Research Centre (AGACAL-CIAM), Unit of Organic Contaminants, Apartado 10, E-15080, A Coruña. Spain.

*Correspondence: [maria.llompart@usc.es](mailto:maria.llompart@usc.es) ;
Tel.: +34-881-814-225

**
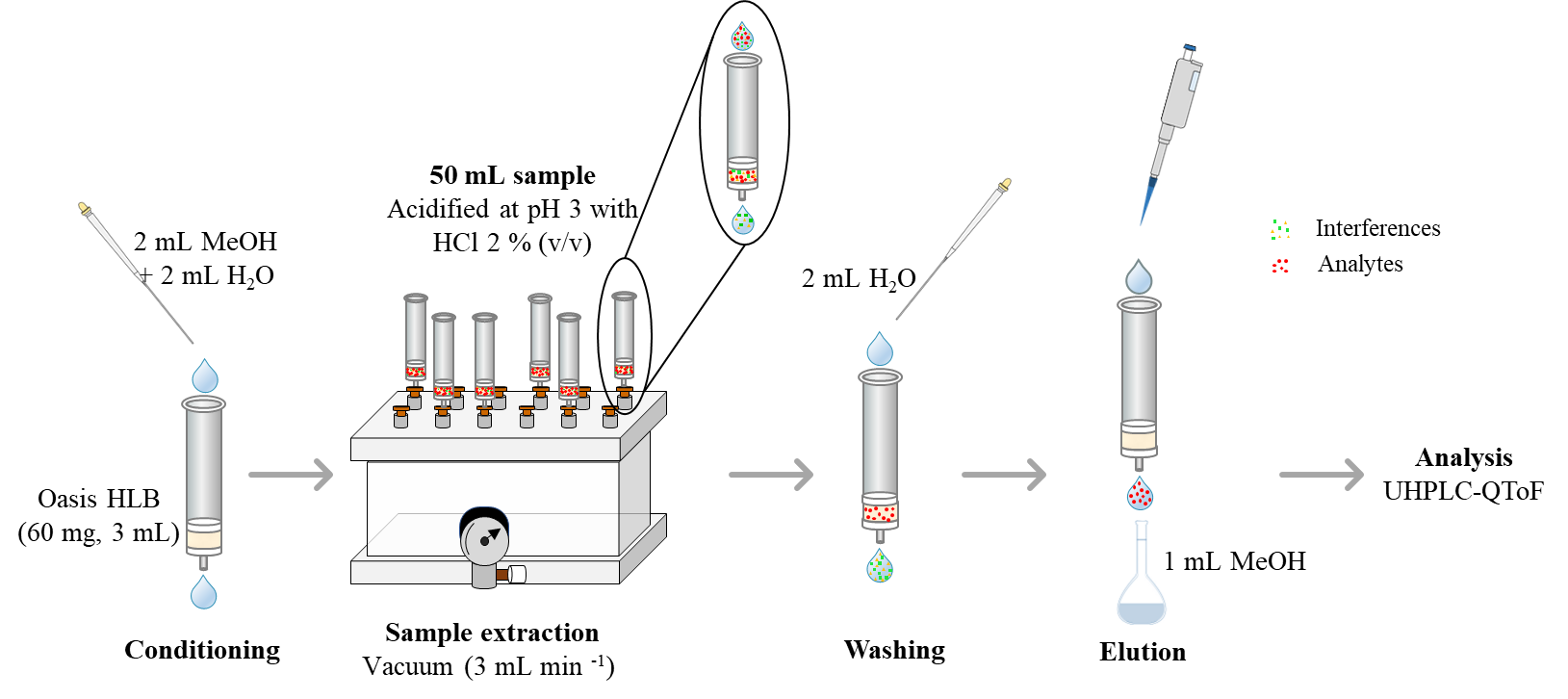
Fig. S1.** Scheme of the sample preparation procedure under optimal conditions.


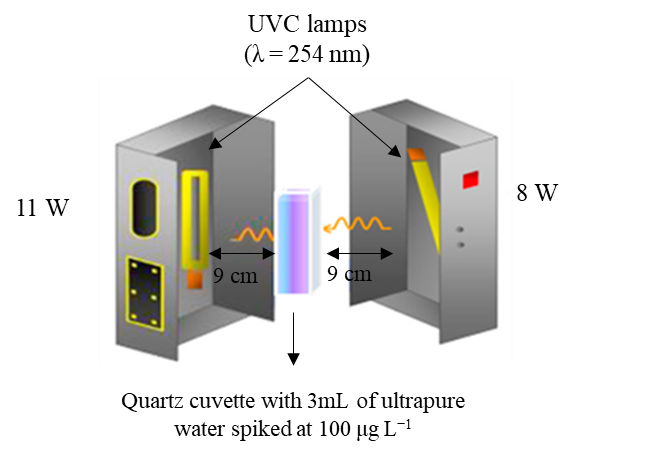


**Fig. S2**. Lab-scale photoreactor employed for the removal of antibiotics.

**
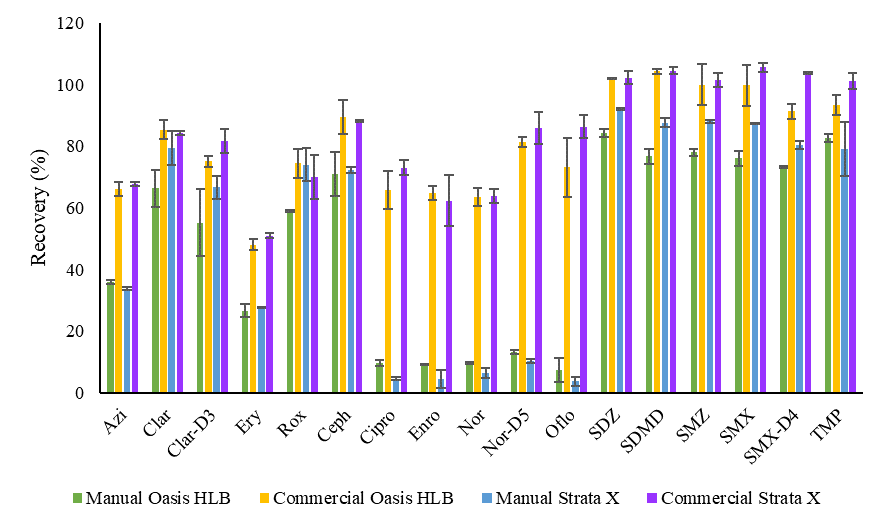
Fig. S3.** Antibiotic recovery comparison between the manual and commercial cartridges for Oasis HLB and Strata X.

**
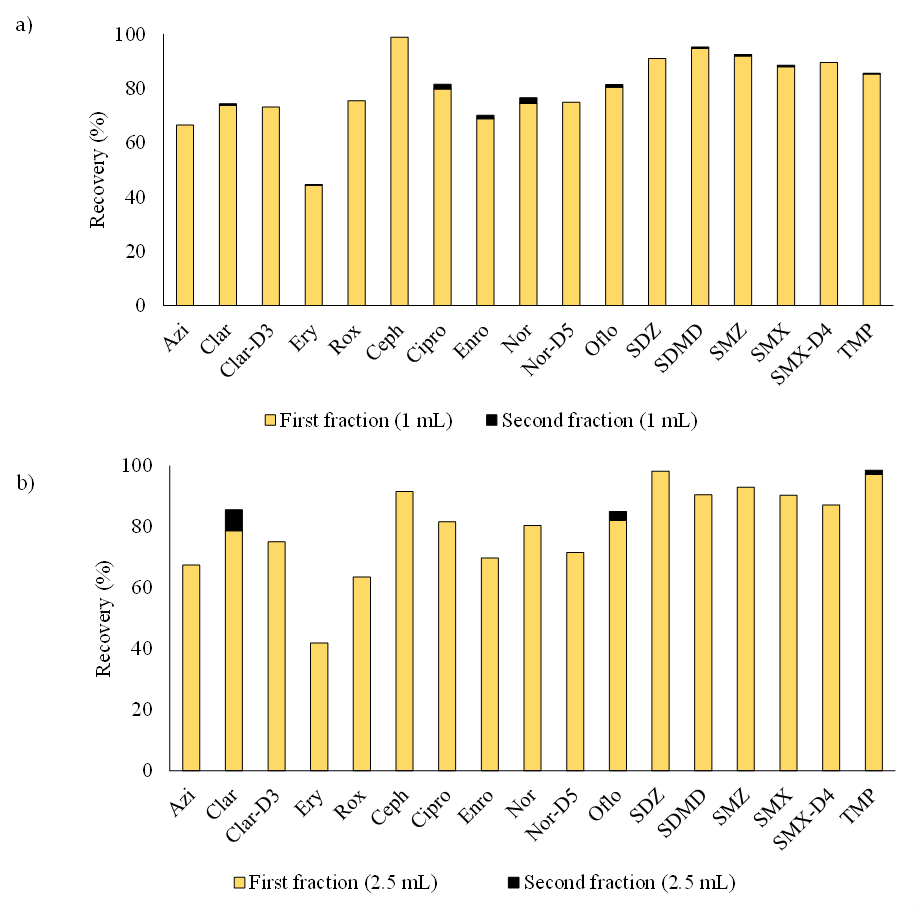
**

**Fig. S4**. Study of the elution volume on SPE efficiency for the target compounds: a) Oasis HLB (60 mg, 3 mL); and b) Oasis HLB (200 mg, 6 mL), respectively.

**
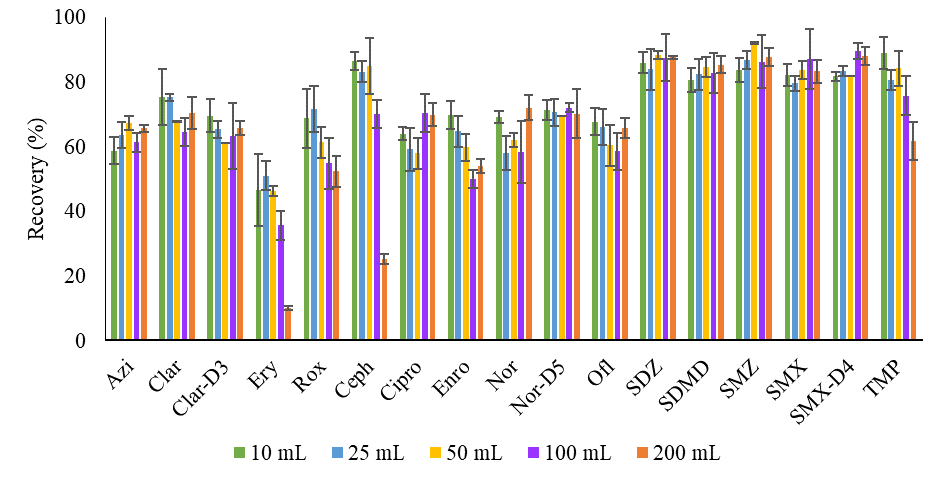
**

**Fig. S5.** Study of the sample breakthrough volume and its influence on SPE antibiotic recoveries.


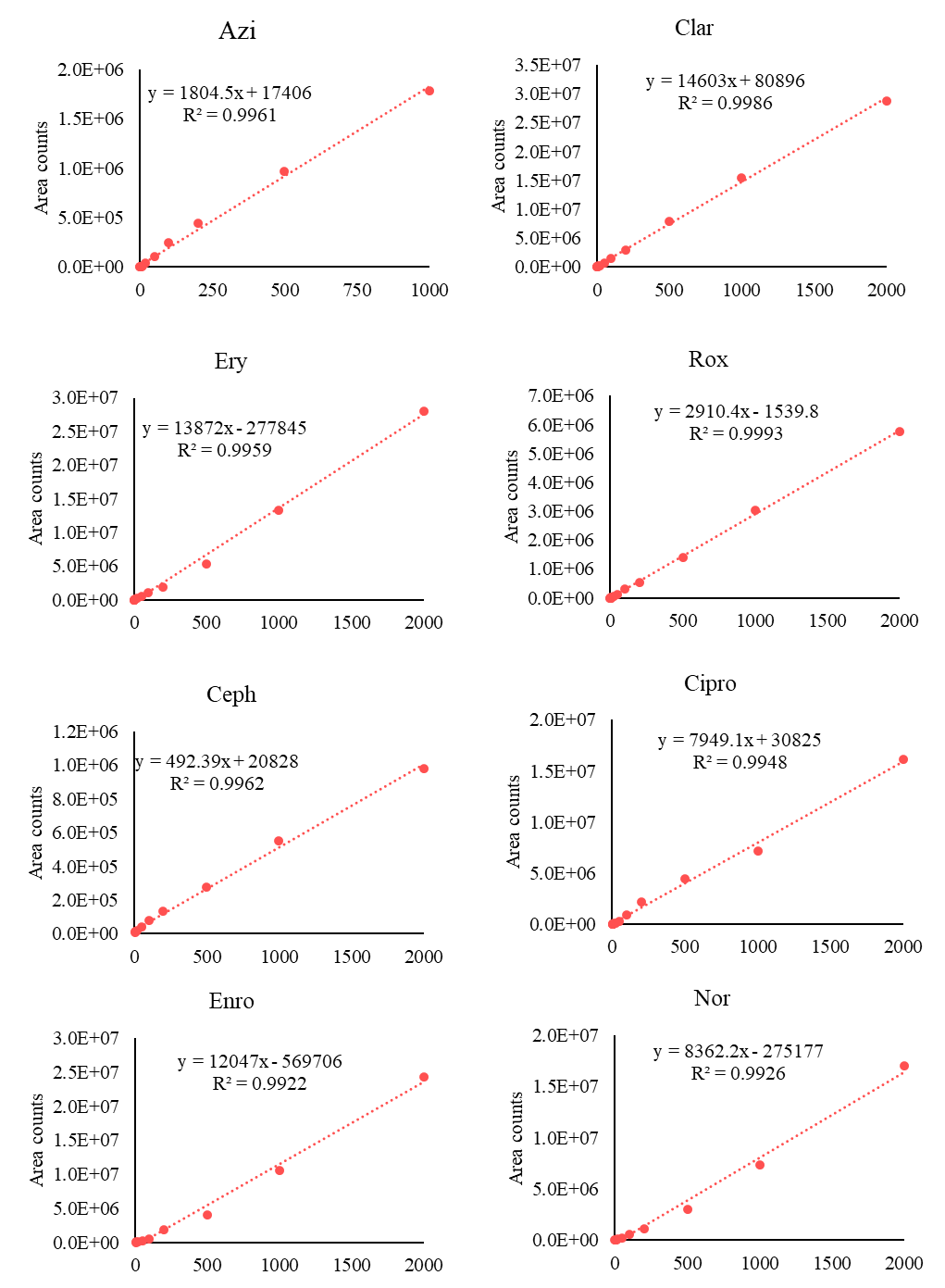


**Fig. S6.** Calibration plots for the target compounds.


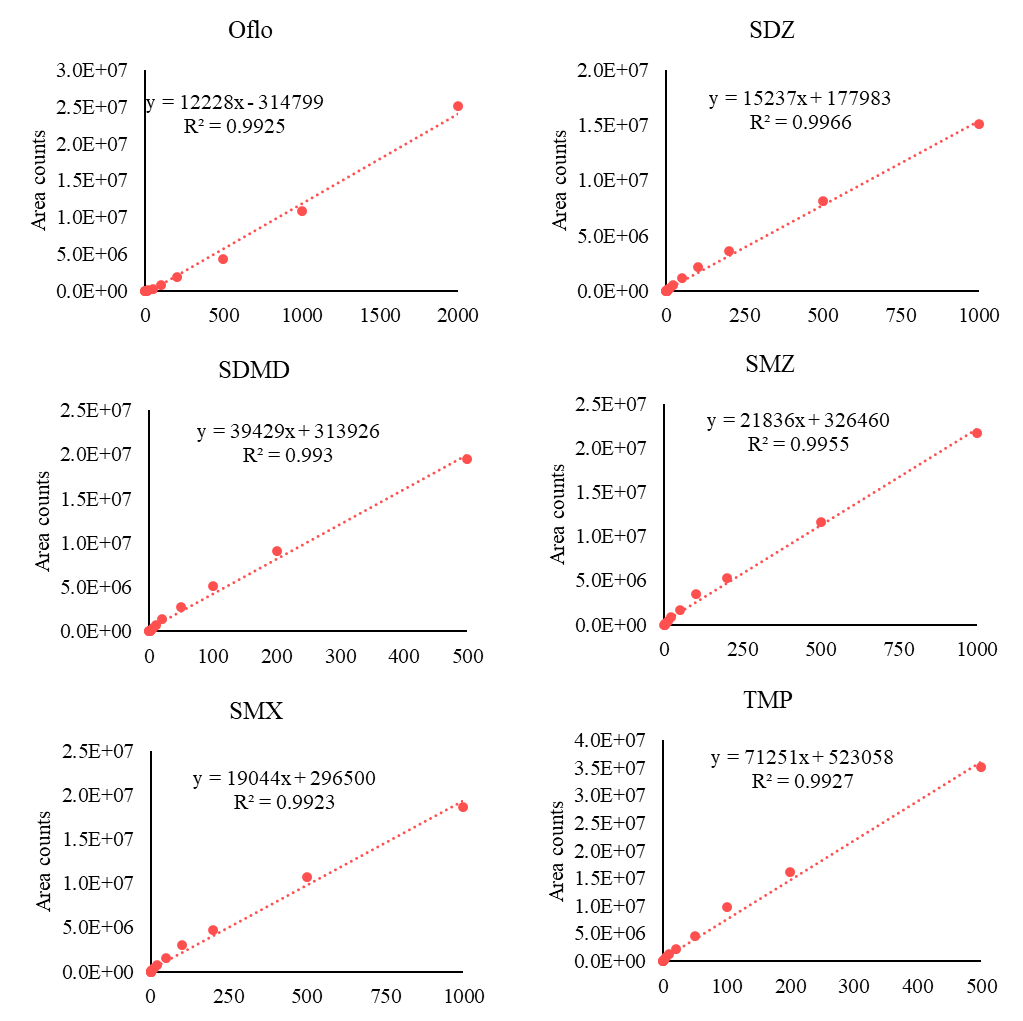


**Fig. S6 (continuation).** Calibration plots for the target compounds.

**Fig. S7**. MS and bbCID MS spectra of the target compounds.

**Fig. S7 (continuation)**. MS and bbCID MS spectra of the target compounds.


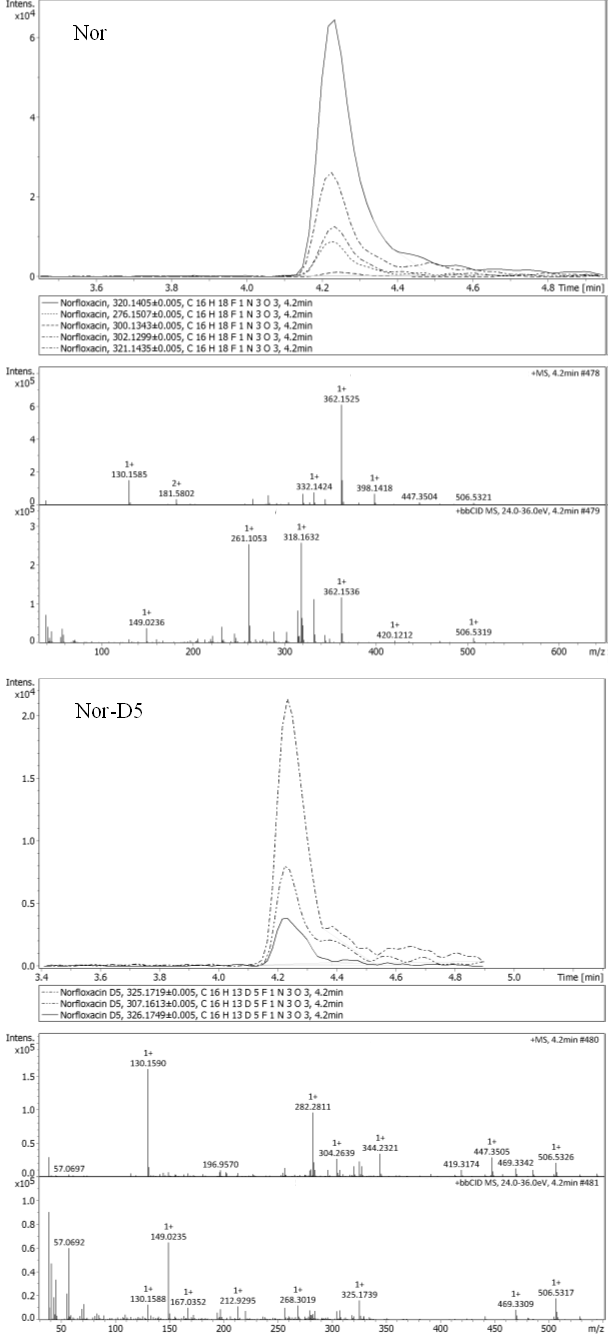


**Fig. S8**. Chromatograms and spectra for Nor and Nor-D5 for the study of the recoveries in ultrapure water spiked at 2 µg L^-1^ employing Oasis HLB (60 mg, 3mL).


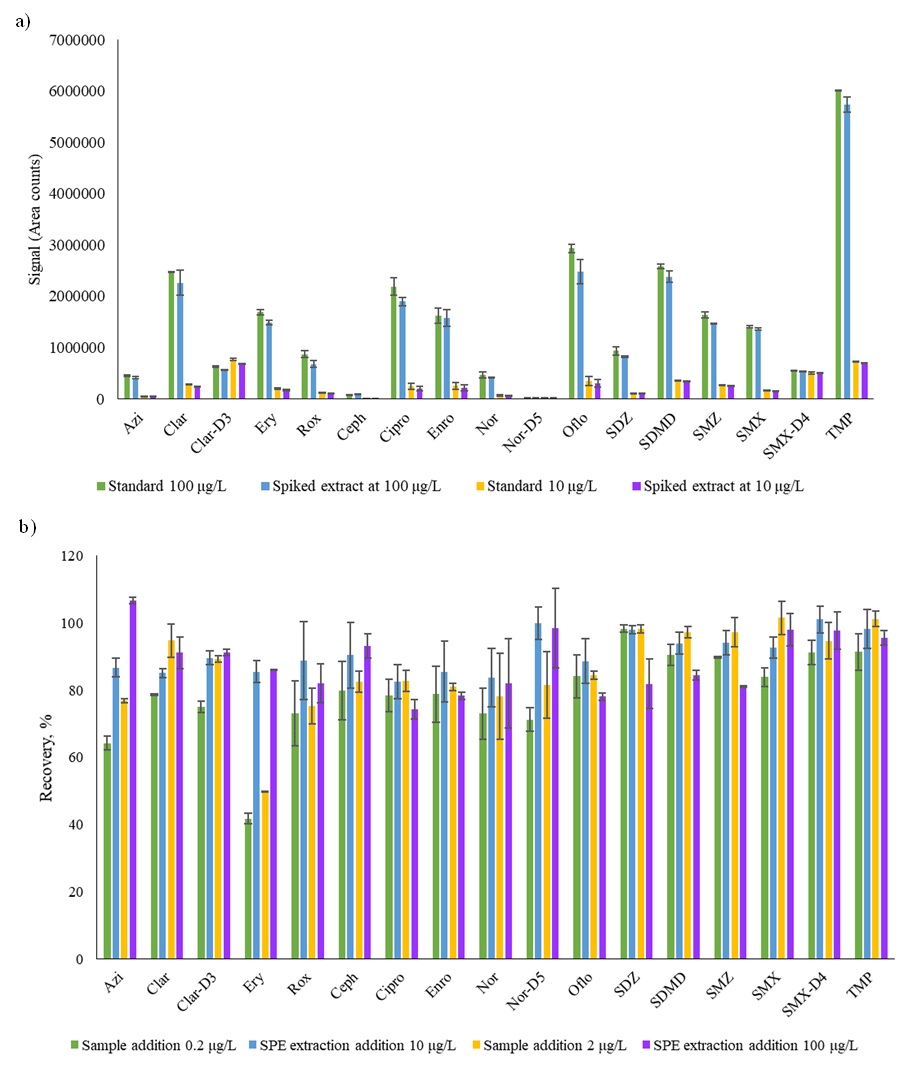
**Fig. S9**. Evaluation of matrix effect into the ionization source: a) comparison of standard solutions and spiked extracts after SPE; b) recovery values between the spiked extracts and the obtained extracts after SPE, using fortified ultrapure water.


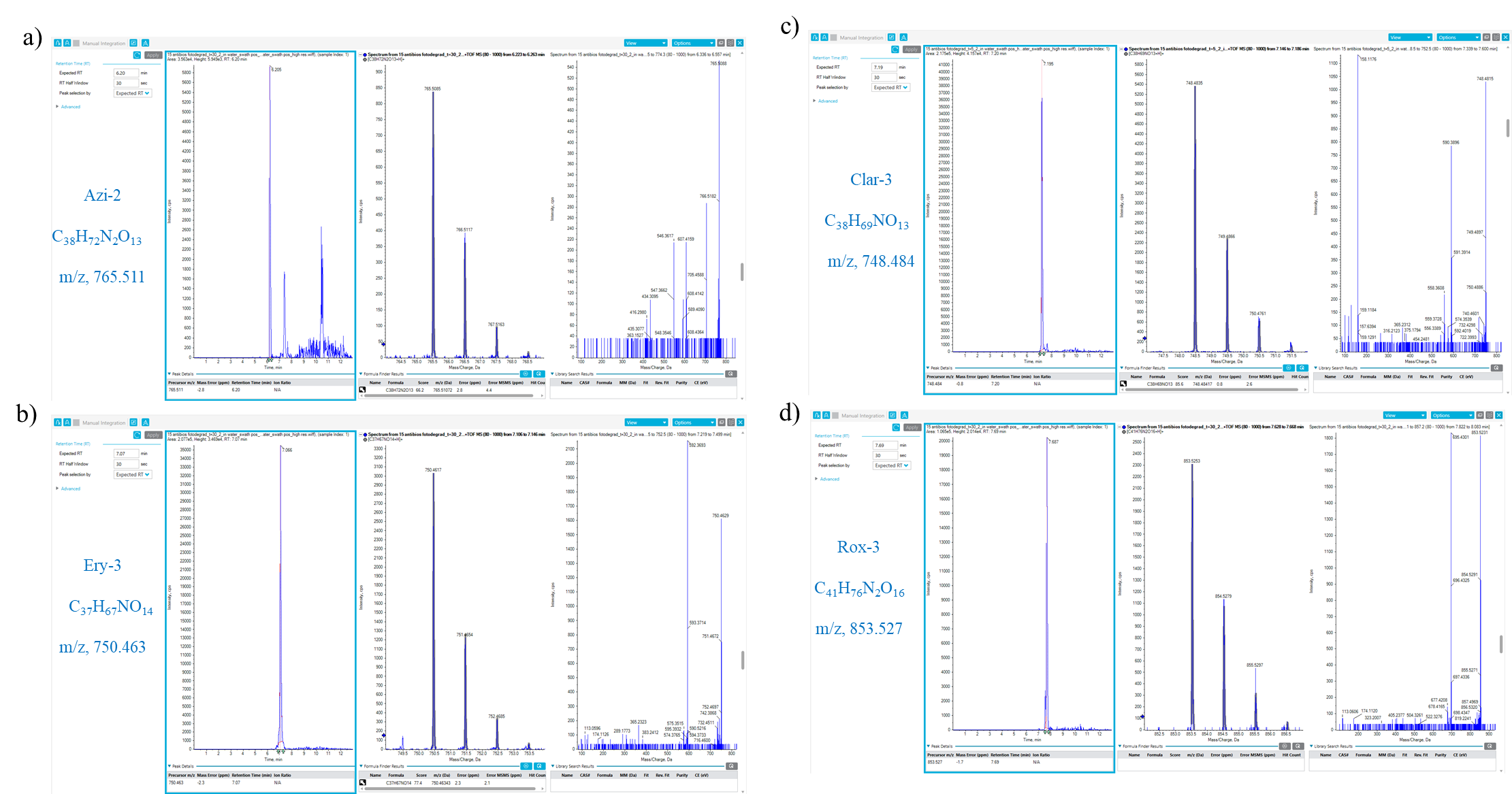
**Fig. S10**. Chromatographic peaks, isotopic profiles and MS/MS spectra of the photodegradation products: a) Azi-2; b) Clar-3; c) Ery-3 and d) Rox-2.

**Table S1.** Gradient elution program for the liquid chromatography separation method.

| **Retention (min)** | **Flow (mL min^-1^)** | **% A^a^** | **% B^b^** |
| --- | --- | --- | --- |
| 0.0 | 0.200 | 96.0 | 4.0 |
| 0.1 | 0.200 | 96.0 | 4.0 |
| 1.0 | 0.200 | 81.7 | 18.3 |
| 2.5 | 0.223 | 50.0 | 50.0 |
| 14.0 | 0.400 | 0.1 | 99.9 |
| 16.0 | 0.480 | 0.1 | 99.9 |
| 16.1 | 0.480 | 96.0 | 4.0 |
| 19.0 | 0.480 | 96.0 | 4.0 |
| 19.1 | 0.200 | 96.0 | 4.0 |
| 20.0 | 0.200 | 96.0 | 4.0 |

^a^A: H_2_O with 0.1 % formic acid and 5 mM ammonium formate; ^b^B: methanol with 0.1 % formic acid and 5 mM ammonium formate.

**Table S2.** Antibiotic concentrations found in real water samples by other authors.

| **Compounds** | **Type of water** | **Concentration (ng L^-1^)** | **Reference** |
| --- | --- | --- | --- |
| 53 antibiotics (Cipro, Enro, Nor, Oflo, SDZ, SMZ, SMX, TMP, Ceph, Azi, Clar, Rox) | EF, INF, HW, River | 19-1036 | Gros et al., 2013 |
| 19 antibiotics (Clar, Ery, Rox, SDZ, SDMD, SMX, Cipro, Enero, Nor, Oflo) | EF, INF | 7.5-1288 | Tylová et al., 2013 |
| 23 drugs (Azi, Clar, Rox, Cipro, SMX, TMP) | EF, INF | 12-8263 | Rossmann et al., 2014 |
| 26 drugs (Cipro, Enro, Nor, Oflo,SDZ, SMZ, SMX TMP) | River water, WWTP and lagoon | 4.2-32.6 | Tlili et al., 2016 |
| 22 pharmaceuticals (Azi, Ery, Clar, Rox, Cipro, Enero, Nor, SDZ, SMZ, SMX, TMP) | EF, INF | 110-4914 | Čizmić et al., 2017 |
| 8 antibiotics and metabolites (SDZ, SDMD, SMZ) | EF, INF | 13.5-124 | Yuan et al., 2019 |
| 8 antibiotics (Cipro, Enro, Nor, Oflo, SDZ, SDMD, SMZ, SMX) | EF, INF | 4.6-2733 | Martínez-Orgániz et al., 2021 |
| 14 antibiotics | EF, INF, river water and well water | 1.6-3500 | This study |

**Table S3.** Non-target compounds identified in real water samples by QToF-HRMS.

| **Compound** | **RT (min)** | **Precursor Mass (m/z)** | **Sample** | **Peak Area** | **Signal Intensity** | **Mass error (ppm)** | **Library score** | **Combined score** | **Isotope Ratio Difference** |
| --- | --- | --- | --- | --- | --- | --- | --- | --- | --- |
| **2,6-Dichlorbenzamide** | 5.61 | 189.982 | EF2 | 169500 | 24190 | -2.2 | 97.6 | 83.0 | 3.8 |
|  |  |  | EF3 | 145000 | 23100 | 0.1 | 97.7 | 66.2 | 6.4 |
|  |  |  | EF4 | 70590 | 10840 | -3.2 | 97.3 | 76.2 | 5.1 |
|  |  |  | INF1 | 70310 | 12330 | -4.5 | 96.8 | 58.0 | 15.1 |
| **2,6-dimethylaniline** | 3.78 | 122.096 | EF2 | 47440 | 1470 | 0.4 | 85.4 | 78.2 | 11.9 |
| **2-Amino-14,16-dimethyloctadecan-3-ol** | 8.58 | 314.342 | EF3 | 174100 | 28830 | 0.5 | 93 | 88.3 | 6.1 |
|  |  |  | HW2 | 133300 | 26920 | 0.4 | 94.2 | 88.0 | 4.5 |
| **4-androstene-3,17-dione/6-dehydrotestosterone** | 8.59 | 287.201 | HW2 | 31680 | 4804 | -2.5 | 96.2 | 70.6 | 9.7 |
| **8-Hydroxyquinoline** | 3.93 | 146.06 | EF2 | 57450 | 7753 | -0.5 | 96.8 | 88.8 | 5.3 |
|  |  |  | EF3 | 73650 | 10700 | -3.8 | 50.6 | 60.8 | 8.7 |
|  |  |  | HW2 | 661300 | 83450 | -1.8 | 97.1 | 87.6 | 3 |
|  |  |  | RW | 9606 | 1180 | -7.2 | 93.4 | 51.7 | 10.7 |
|  |  |  | WW | 16650 | 2590 | -7.2 | 97.6 | 57.2 | 5.2 |
| **Acetaminophen** | 2.13 | 152.071 | HW1 | 185500 | 19890 | -3.4 | 91.3 | 59.1 | 4.1 |
|  |  |  | HW2 | 122000 | 11260 | -1.4 | 86.5 | 61.8 | 5.1 |
|  |  |  | INF1 | 3034000 | 260100 | -1.4 | 97 | 87.4 | 1.9 |
|  |  |  | RW | 31530 | 3103 | -3.6 | 69.4 | 71.8 | 1.7 |
| **Acetanilide** | 4.52 | 136.076 | INF1 | 20520 | 2729 | 1.5 | 100 | 69.0 | 95.5 |
| **Adenine** | 0.91 | 136.062 | INF1 | 69310 | 9077 | -1 | 99.6 | 90.5 | 1.3 |
| **Altenuene** | 6.88 | 293.102 | EF4 | 24520 | 4180 | 5.9 | 99.6 | 50.5 | 39.8 |
| **Amisulpride** | 5.14 | 370.18 | EF1 | 862300 | 129200 | -0.8 | 100 | 93.2 | 2.3 |
|  |  |  | EF4 | 652900 | 97400 | -0.2 | 100 | 95.3 | 1.9 |
|  |  |  | EF5 | 1093000 | 164200 | -2.7 | 100 | 80.4 | 4.3 |
|  |  |  | HW1 | 137700 | 21840 | 0.3 | 99.1 | 82.2 | 11.7 |
|  |  |  | HW2 | 404100 | 59660 | 0.4 | 100 | 86.4 | 7.3 |
|  |  |  | INF1 | 479900 | 69460 | -0.7 | 100 | 92.1 | 3.1 |
| **Arecoline** | 4.32 | 156.102 | EF3 | 39620 | 5317 | 0.3 | 98.7 | 72.5 | 1.1 |
| **Atorvastatin** | 7.97 | 559.26 | EF1 | 82050 | 13760 | 0.6 | 100 | 85.3 | 9.1 |
|  |  |  | EF2 | 32900 | 5762 | -0.9 | 89.7 | 82.1 | 7.7 |
|  |  |  | EF3 | 20360 | 3361 | 0.4 | 83.8 | 67.1 | 37 |
|  |  |  | EF4 | 69900 | 11570 | 0.4 | 100 | 80.8 | 12.6 |
|  |  |  | HW2 | 32120 | 6669 | -0.1 | 94.6 | 76.5 | 15.7 |
|  |  |  | INF1 | 72500 | 13280 | -0.9 | 100 | 77.9 | 13 |
| **Benzydamine** | 7.1 | 310.191 | HW2 | 1240000 | 198200 | 0.3 | 97.8 | 95.8 | 0.9 |
| **β-Ergocryptine** | 7.14 | 576.318 | EF5 | 1515000 | 216500 | -1.2 | 96 | 80.0 | 9.7 |
|  |  |  | HW1 | 106500 | 17330 | 3.9 | 51.8 | 61.6 | 7.7 |
|  |  |  | RW | 1466000 | 232200 | 3.4 | 74.1 | 66.6 | 7.7 |
|  |  |  | WW | 376200 | 53060 | 3.6 | 60.9 | 66.0 | 4.6 |
| **Bethanidine** | 4.4 | 178.134 | EF1 | 152200 | 23230 | -2.9 | 85.2 | 74.2 | 7.1 |
|  |  |  | EF3 | 44110 | 5600 | -1.2 | 86.8 | 69.1 | 17.9 |
|  |  |  | EF4 | 456000 | 67470 | -1.9 | 86.1 | 84.1 | 2.9 |
|  |  |  | HW1 | 260800 | 35610 | -1.5 | 85.8 | 83.9 | 4.6 |
|  |  |  | HW2 | 84110 | 13210 | -3.6 | 84.5 | 76.8 | 1.2 |
|  |  |  | INF1 | 579000 | 83640 | -2.5 | 85.9 | 82.4 | 1.8 |
| **Bezafibrate** | 7.27 | 362.115 | HW1 | 80070 | 14220 | -0.8 | 96 | 77.0 | 13.3 |
| **Bisoprolol** | 6.15 | 326.233 | EF5 | 344000 | 57710 | -5.8 | 99.1 | 56.8 | 2.8 |
|  |  |  | HW1 | 336000 | 45570 | -0.7 | 99.4 | 59.1 | 11.1 |
| **Boldenone** | 8.59 | 287.201 | HW2 | 31680 | 4804 | -2.5 | 54.4 | 60.2 | 9.7 |
| **Brucine** | 8.00 | 395.197 | WW | 39370 | 5363 | 0.8 | 97.9 | 80.8 | 10.1 |
| **Caffeine** | 5.32 | 195.088 | EF1 | 137900 | 18150 | -0.5 | 83.3 | 80.5 | 8.4 |
|  |  |  | EF2 | 87480 | 13260 | -3 | 93.9 | 78.8 | 4 |
|  |  |  | EF3 | 292300 | 38820 | -0.4 | 96.2 | 95.3 | 0.9 |
|  |  |  | EF4 | 162100 | 24380 | -1.8 | 79.4 | 80.0 | 2.4 |
|  |  |  | EF5 | 195400 | 33580 | -1.6 | 97.3 | 61.0 | 7.4 |
|  |  |  | HW1 | 16490000 | 2086000 | -0.9 | 98.4 | 93.5 | 0.4 |
|  |  |  | HW2 | 12880000 | 1824000 | 0.5 | 98.9 | 95.0 | 0.5 |
|  |  |  | INF1 | 5563000 | 699700 | -1.3 | 98.3 | 89.1 | 1.6 |
|  |  |  | RW | 94500 | 16690 | -0.1 | 76.4 | 66.7 | 1.8 |
| **Candesartan** | 7.58 | 441.167 | EF3 | 99990 | 16920 | -3.1 | 87.2 | 75.2 | 4.6 |
|  |  |  | EF4 | 44030 | 6331 | -0.6 | 76.9 | 69.1 | 14.9 |
|  |  |  | HW2 | 84020 | 14370 | -0.1 | 77.8 | 85.1 | 4.9 |
| **Carbamazepine** | 7.02 | 237.102 | EF1 | 93180 | 13710 | -0.8 | 95.7 | 71.5 | 17.8 |
|  |  |  | EF2 | 21080 | 3427 | 1.7 | 91.7 | 67.7 | 18 |
|  |  |  | EF4 | 65430 | 10520 | -0.5 | 88.4 | 85.0 | 6.8 |
|  |  |  | HW2 | 25350 | 5385 | -2 | 50 | 55.0 | 19.5 |
| **Carbamazepine 10,11-epoxide** | 5.69 | 253.097 | EF1 | 184400 | 26330 | -0.9 | 97.3 | 86.6 | 4.3 |
|  |  |  | EF4 | 163100 | 23230 | -0.8 | 95.2 | 91.5 | 1.9 |
|  |  |  | EF5 | 499200 | 76060 | -0.9 | 98 | 84.3 | 5.7 |
|  |  |  | INF1 | 74610 | 16000 | -0.6 | 91.3 | 81.0 | 8.9 |
| **Cetirizine** | 7.43 | 389.163 | EF1 | 103100 | 17720 | -0.9 | 98.6 | 61.5 | 8.8 |
|  |  |  | EF3 | 81660 | 13210 | 0.7 | 98.6 | 69.5 | 2.6 |
|  |  |  | EF4 | 86290 | 12280 | 0.6 | 98.7 | 84.8 | 7.5 |
|  |  |  | HW1 | 334900 | 50060 | -0.5 | 98.3 | 88.5 | 5.9 |
|  |  |  | HW2 | 173300 | 29470 | -0.6 | 98.5 | 89.7 | 3.8 |
| **Cetobemidone** | 4.71 | 248.165 | WW | 58280 | 7664 | -4.1 | 76.6 | 56.6 | 1.8 |
| **Chlorbenzoxamine** | 6.18 | 435.22 | HW2 | 125400 | 15840 | 0.7 | 83 | 58.3 | 8.5 |
| **Chlorfenvinphos** | 8.24 | 358.977 | RW | 60750 | 9495 | 0.5 | 100 | 94.2 | 1.6 |
| **Citalopram** | 6.44 | 325.171 | EF1 | 180600 | 30870 | -3.4 | 100 | 59.2 | 34.6 |
|  |  |  | HW1 | 292200 | 49020 | 0.9 | 100 | 84.8 | 7.2 |
|  |  |  | HW2 | 320500 | 53910 | 3.9 | 98.3 | 54.8 | 22.9 |
|  |  |  | INF1 | 103700 | 14930 | -3.2 | 100 | 59.7 | 27.1 |
| **Clozapine** | 6.3 | 327.137 | EF3 | 29120 | 4554 | 2.4 | 91.4 | 60.7 | 34.1 |
|  |  |  | HW1 | 1792000 | 306800 | 0.3 | 99.4 | 90.7 | 5.9 |
| **Coumarin** | 6.41 | 147.044 | HW2 | 86040 | 13060 | 0 | 93.5 | 71.1 | 52 |
| **Crotetamide** | 5.94 | 227.175 | HW1 | 350500 | 50200 | -3.1 | 99.6 | 82.8 | 1.5 |
| **Cyanophos** | 5.00 | 244.019 | HW2 | 3088 | 639 | 0.5 | 73.8 | 66.2 | 119.4 |
| **Dapiprazole** | 6.15 | 326.234 | HW1 | 323400 | 45070 | -3.1 | 84.4 | 53.5 | 22.7 |
| **DEET** | 7.24 | 192.138 | EF1 | 277800 | 43980 | -2.1 | 82.1 | 83.8 | 0.4 |
|  |  |  | EF2 | 127600 | 18440 | -1.4 | 82.5 | 61.6 | 4.4 |
|  |  |  | EF3 | 182400 | 30960 | -2.8 | 82.4 | 61.2 | 1.8 |
|  |  |  | EF4 | 111200 | 16940 | -0.3 | 67.1 | 82.0 | 2.3 |
|  |  |  | HW1 | 54580 | 11820 | 0.8 | 81.8 | 75.3 | 11.2 |
|  |  |  | HW2 | 37450 | 7402 | 0.3 | 67.1 | 72.9 | 11.3 |
|  |  |  | INF1 | 38000 | 7106 | 0.5 | 86.8 | 64.5 | 4.7 |
|  |  |  | WW | 48020 | 8058 | -2 | 83.8 | 53.5 | 10 |
| **Desmethylcitalopram** | 6.44 | 311.155 | EF4 | 63550 | 9766 | -3.2 | 98.7 | 58.9 | 49.8 |
|  |  |  | HW1 | 83060 | 14850 | -2.5 | 100 | 61.5 | 33.1 |
| **Diclofenac** | 7.95 | 296.024 | EF1 | 42950 | 9974 | 0.9 | 100 | 92.5 | 1.9 |
|  |  |  | EF2 | 16910 | 3379 | 4.5 | 97.7 | 68.2 | 7.3 |
|  |  |  | EF3 | 106500 | 18120 | 0.5 | 100 | 89.7 | 4.6 |
|  |  |  | EF4 | 30590 | 6239 | 0.6 | 100 | 83.7 | 9.7 |
|  |  |  | EF5 | 274300 | 52150 | -4.5 | 100 | 74.8 | 2.8 |
|  |  |  | INF1 | 12610 | 3726 | 5.2 | 97.9 | 64.6 | 10.6 |
| **Diltiazem** | 7.25 | 415.169 | EF1 | 91180 | 13960 | -0.5 | 92.6 | 60.5 | 9.2 |
|  |  |  | EF3 | 16100 | 3015 | 1.1 | 84.3 | 65.5 | 23.3 |
|  |  |  | EF4 | 79210 | 13660 | 1.8 | 95.2 | 64.6 | 26.9 |
|  |  |  | HW1 | 845300 | 138600 | -0.7 | 100 | 94.2 | 1.1 |
|  |  |  | HW2 | 5700000 | 858400 | 0.1 | 100 | 96.9 | 1.3 |
| **Dimethoate** | 5.47 | 230.007 | EF1 | 276800 | 38350 | -3.2 | 99.4 | 83.0 | 1.8 |
| **Dodemorph** | 8.71 | 282.279 | EF4 | 368500 | 68120 | -1.8 | 75.5 | 82.6 | 2.2 |
|  |  |  | HW1 | 481900 | 90670 | -0.4 | 77.8 | 89.0 | 2.2 |
|  |  |  | HW2 | 666500 | 123900 | -1.5 | 73.8 | 83.2 | 2.2 |
|  |  |  | INF1 | 686900 | 147100 | -0.2 | 80.4 | 91.1 | 0.3 |
| **EDDP** | 7.16 | 278.19 | EF4 | 140000 | 21240 | -1 | 95.7 | 78.9 | 10.5 |
|  |  |  | HW1 | 375400 | 55490 | 0.4 | 96.6 | 90.0 | 5.2 |
|  |  |  | INF1 | 112300 | 21560 | -0.8 | 99.1 | 69.4 | 26.4 |
| **Emetine** | 9.11 | 481.306 | EF1 | 13830 | 1602 | -0.1 | 100 | 74.7 | 52.9 |
| **Eprosartan** | 6.65 | 425.153 | EF1 | 1222000 | 162000 | -2.3 | 100 | 84.9 | 2.1 |
|  |  |  | EF2 | 2262000 | 327200 | -1.5 | 100 | 91.8 | 1.1 |
|  |  |  | EF4 | 483900 | 70760 | 0.6 | 78.5 | 88.5 | 0.8 |
|  |  |  | HW1 | 118100 | 18380 | -0.3 | 98 | 90.4 | 5.1 |
|  |  |  | INF1 | 1117000 | 129700 | -1 | 100 | 85.4 | 5.9 |
| **Ergosine** | 6.23 | 548.287 | WW | 17590 | 2027 | 0.8 | 84.4 | 66.1 | 34.9 |
| **estr-4-ene-3,17-dione** | 8.67 | 273.185 | EF1 | 573700 | 110700 | 0.2 | 71.3 | 65.9 | 1.2 |
|  |  |  | EF2 | 506400 | 97140 | -0.3 | 68.7 | 63.3 | 2.4 |
|  |  |  | EF3 | 419300 | 80690 | -2.5 | 73.8 | 59.4 | 2.1 |
|  |  |  | EF4 | 327900 | 69860 | -0.5 | 68.3 | 84.6 | 3.6 |
|  |  |  | INF1 | 240100 | 43170 | -0.6 | 69.3 | 59.0 | 5.4 |
|  |  |  | RW | 423700 | 78970 | -0.8 | 67.8 | 53.8 | 8.9 |
| **Fenbufen** | 7.46 | 255.102 | EF1 | 175500 | 34410 | -0.6 | 72.2 | 51.8 | 11.9 |
|  |  |  | EF2 | 38210 | 5583 | 0.9 | 98.8 | 64.0 | 6.8 |
|  |  |  | EF3 | 165400 | 28100 | -1.7 | 86.7 | 62.0 | 4.4 |
|  |  |  | EF4 | 152800 | 24990 | 0.7 | 97.2 | 68.5 | 3.3 |
|  |  |  | HW1 | 1051000 | 165900 | -0.7 | 67.8 | 83.5 | 3.4 |
|  |  |  | HW2 | 1227000 | 207200 | 0.3 | 86.4 | 93.5 | 0.6 |
|  |  |  | INF1 | 133100 | 21600 | -0.6 | 85.4 | 75.5 | 12.7 |
| **Fenofibric acid** | 8.01 | 319.073 | EF1 | 132900 | 25540 | -1 | 99.2 | 87.3 | 6.1 |
|  |  |  | EF2 | 62050 | 11040 | 0.4 | 98.5 | 92.0 | 2.6 |
|  |  |  | EF3 | 120500 | 20150 | 0.5 | 98.6 | 93.8 | 1.5 |
|  |  |  | EF4 | 96080 | 16970 | 0.4 | 99.1 | 93.2 | 3.2 |
|  |  |  | EF5 | 123800 | 22860 | -4.5 | 100 | 73.2 | 2.9 |
|  |  |  | HW1 | 341900 | 60230 | -0.2 | 99.2 | 93.2 | 2.5 |
|  |  |  | HW2 | 45020 | 9081 | -1.2 | 97.9 | 64.1 | 5.9 |
|  |  |  | INF1 | 88500 | 20780 | 0.1 | 99.1 | 89.8 | 6 |
| **Fenuron** | 5.01 | 165.102 | EF1 | 7415000 | 901700 | -3.4 | 57.4 | 70.0 | 1.5 |
| **Fexofenadine** | 7.21 | 502.295 | EF3 | 20020 | 3227 | -0.1 | 97.3 | 73.7 | 40.1 |
| **Flecainide** | 6.2 | 415.145 | EF1 | 365300 | 62450 | -1.7 | 99.2 | 85.6 | 3.6 |
|  |  |  | EF2 | 235200 | 34100 | -3 | 99.4 | 73.8 | 7.6 |
|  |  |  | EF3 | 903200 | 116000 | 0.6 | 99.2 | 93.5 | 1.8 |
|  |  |  | EF4 | 373100 | 46720 | -0.9 | 99.4 | 85.2 | 7.7 |
|  |  |  | EF5 | 778400 | 129400 | -5.3 | 99.4 | 61.2 | 9.1 |
|  |  |  | HW1 | 194300 | 21130 | 1.3 | 96.9 | 78.0 | 11.1 |
|  |  |  | HW2 | 753200 | 108200 | -0.9 | 99.5 | 90.1 | 4.2 |
|  |  |  | INF1 | 193800 | 30050 | 0.6 | 99.3 | 78.4 | 13.7 |
| **Fluconazole** | 5.52 | 307.111 | EF1 | 34870 | 5151 | 2.4 | 95.3 | 66.9 | 13.3 |
|  |  |  | HW1 | 130500 | 18750 | -0.2 | 99.8 | 72.6 | 1.5 |
| **Fludrocortisone Acetate** | 5.00 | 423.218 | EF1 | 513000 | 59400 | 3.5 | 100 | 60.7 | 4.3 |
|  |  |  | EF5 | 203600 | 16730 | -0.3 | 100 | 68.8 | 4.4 |
|  |  |  | HW1 | 5022000 | 345100 | 3 | 100 | 74.4 | 7.9 |
|  |  |  | HW2 | 2301000 | 217700 | 3.7 | 100 | 75.7 | 3.7 |
| **Hordenine** | 4.37 | 166.123 | HW2 | 66380 | 9482 | -2.6 | 97.4 | 64.6 | 2.5 |
| **Irbesartan** | 7.79 | 429.24 | EF1 | 503300 | 84770 | -0.6 | 96 | 86.6 | 6.8 |
|  |  |  | EF2 | 226300 | 38430 | -0.3 | 97.2 | 89.3 | 6.4 |
|  |  |  | EF3 | 272200 | 43840 | -0.7 | 96.8 | 83.1 | 8.3 |
|  |  |  | EF4 | 377000 | 64350 | 0.2 | 96.1 | 93.2 | 3.2 |
|  |  |  | EF5 | 2989000 | 490500 | -7.3 | 96.9 | 62.1 | 2.5 |
|  |  |  | HW1 | 280500 | 48480 | -0.3 | 96.4 | 85.2 | 9.4 |
|  |  |  | INF1 | 290200 | 58070 | -0.1 | 96.9 | 92.8 | 3.9 |
| **Kavain** | 7.44 | 231.102 | HW1 | 987100 | 159400 | 0.2 | 72.5 | 88.8 | 2.1 |
| **Ketocaine** | 6.21 | 292.227 | EF2 | 46920 | 7378 | -1.9 | 82.3 | 77.7 | 6 |
| **Levocabastine** | 8.3 | 421.229 | EF5 | 168400 | 29120 | -1 | 95.3 | 73.9 | 15.8 |
|  |  |  | RW | 41690 | 8261 | -1.7 | 93 | 60.4 | 97.8 |
| **Lidocaine** | 4.71 | 235.18 | EF3 | 761500 | 109700 | -0.9 | 99.7 | 92.3 | 2.3 |
|  |  |  | HW1 | 153100 | 25400 | -0.8 | 99.7 | 52.6 | 16.2 |
|  |  |  | HW2 | 486600 | 73880 | -0.9 | 99.7 | 89.0 | 3.3 |
| **Lolitrem B** | 7.9 | 686.405 | HW1 | 81600 | 10480 | 3.5 | 95.9 | 62.4 | 15.2 |
| **Losartan** | 7.42 | 423.169 | EF1 | 293600 | 42820 | -0.3 | 99.7 | 89.9 | 4.8 |
|  |  |  | EF3 | 242100 | 38280 | -2.1 | 99.5 | 88.5 | 0.4 |
|  |  |  | EF4 | 243400 | 39800 | -0.3 | 99.7 | 86.6 | 7.6 |
|  |  |  | HW1 | 177300 | 29910 | -1.2 | 91.3 | 90.2 | 0.9 |
|  |  |  | HW2 | 82200 | 14370 | 0.7 | 97.3 | 83.5 | 8.4 |
|  |  |  | INF1 | 239800 | 36520 | -0.8 | 99.6 | 92.9 | 1.7 |
| **Mefloquine** | 6.46 | 379.124 | EF4 | 10800 | 2063 | 4.1 | 99.5 | 58.7 | 17.1 |
| **Mepivacaine** | 4.76 | 247.18 | EF1 | 32190 | 3574 | 0.1 | 100 | 63.1 | 9.4 |
|  |  |  | HW1 | 60520 | 6677 | -1.1 | 100 | 70.5 | 31.3 |
|  |  |  | HW2 | 76080 | 12850 | -2.1 | 100 | 55.4 | 11.6 |
|  |  |  | INF1 | 17200 | 1986 | -0.8 | 100 | 71.6 | 27.2 |
| **Methadone** | 7.43 | 310.217 | HW1 | 2088000 | 328600 | -0.6 | 94.6 | 89.4 | 3.3 |
| **Methcathinone** | 3.72 | 164.107 | HW1 | 17420 | 2682 | -0.2 | 74.5 | 67.9 | 50.3 |
| **Methoxyphenamine** | 5.06 | 180.138 | INF1 | 13770 | 1505 | -1.2 | 80.8 | 51.6 | 12.5 |
| **Moxisylyte** | 4.46 | 280.191 | EF2 | 45950 | 7838 | -0.9 | 59.2 | 83.4 | 2.2 |
|  |  |  | EF4 | 118200 | 18000 | 0.4 | 96.6 | 94.1 | 2.8 |
|  |  |  | HW1 | 299800 | 48770 | -0.3 | 94.8 | 96.3 | 1 |
|  |  |  | INF1 | 104600 | 15270 | -1.7 | 93.2 | 63.9 | 4.1 |
| **Oxazepam** | 7.32 | 287.058 | EF1 | 53030 | 9163 | -2.1 | 94.8 | 82.4 | 4.5 |
|  |  |  | EF5 | 123000 | 24610 | -3.8 | 100 | 76.5 | 3.6 |
| **Oxcarbazepine** | 6.58 | 253.097 | EF1 | 137000 | 18470 | 0.7 | 86.1 | 90.1 | 1.8 |
|  |  |  | EF3 | 30230 | 3770 | 3.3 | 89.9 | 50.5 | 11 |
|  |  |  | EF4 | 106100 | 14980 | 1.2 | 97.2 | 89.2 | 1.8 |
|  |  |  | EF5 | 746400 | 121500 | -3.6 | 98.5 | 77.2 | 1.8 |
| **Paracetamol** | 2.14 | 152.071 | EF4 | 7525 | 914 | -2.4 | 57.7 | 50.1 | 6.7 |
|  |  |  | HW1 | 185500 | 19890 | -3.4 | 99.7 | 61.3 | 4.1 |
|  |  |  | HW2 | 122000 | 11260 | -1.4 | 99.6 | 65.1 | 5.1 |
|  |  |  | INF1 | 3034000 | 260100 | -1.4 | 99.6 | 88.1 | 1.9 |
|  |  |  | RW | 31490 | 3102 | -3.6 | 89.9 | 76.9 | 1.7 |
| **Paraxanthine** | 3.68 | 181.072 | EF4 | 75490 | 8922 | -0.6 | 83.9 | 67.7 | 22.6 |
|  |  |  | EF5 | 487200 | 67100 | -5.4 | 69.6 | 62.0 | 3.8 |
| **Paspalitrem A** | 7.21 | 502.295 | EF3 | 20020 | 3227 | -0.1 | 83.9 | 70.3 | 40.1 |
| **PMA** | 4.37 | 166.123 | HW2 | 66760 | 9502 | -2.6 | 86.4 | 61.9 | 2.5 |
| **PMMA** | 4.23 | 180.138 | EF5 | 20020 | 2788 | -4.3 | 96.4 | 53.6 | 7.9 |
| **Pyroquilon** | 3.93 | 174.091 | EF4 | 102700 | 13840 | -1.7 | 70.6 | 79.7 | 3.7 |
| **Quinine** | 6.16 | 325.191 | EF1 | 122500 | 16370 | 3.2 | 92.7 | 72.3 | 9.3 |
|  |  |  | EF2 | 61000 | 9421 | 0.9 | 71.9 | 81.0 | 3.7 |
| **Rifampicin** | 7.9 | 823.412 | HW1 | 37500 | 6480 | -2.7 | 75.1 | 72.5 | 6.3 |
| **Salicylamide** | 3.82 | 138.055 | HW1 | 23430 | 3750 | -1.2 | 57.3 | 54.0 | 6 |
| **Serotonin** | 3.79 | 177.102 | EF3 | 6303 | 655 | 1.3 | 97.9 | 69.2 | 143.2 |
| **Spinetoram A** | 8.23 | 748.499 | EF1 | 136900 | 9082 | 1.3 | 95.1 | 82.2 | 5.6 |
|  |  |  | EF3 | 108400 | 9076 | 0.3 | 98.7 | 79.0 | 12.7 |
|  |  |  | EF4 | 83750 | 5229 | 3.3 | 64.7 | 50.5 | 21 |
| **Spinosyn A** | 8.26 | 732.468 | EF4 | 36600 | 5096 | 2.4 | 97 | 70.2 | 12.3 |
| **Spinosyn D** | 8.34 | 746.484 | EF5 | 865100 | 102200 | -0.3 | 68.3 | 72.1 | 12.8 |
| **Sulfapyridine** | 4.75 | 250.064 | EF1 | 45150 | 6347 | -0.9 | 96.7 | 90.1 | 2.3 |
|  |  |  | EF5 | 165800 | 27830 | -4.1 | 97.6 | 62.8 | 12.5 |
| **Sulpiride** | 3.69 | 342.148 | EF1 | 610100 | 92740 | 0.8 | 100 | 94.3 | 0.6 |
|  |  |  | EF2 | 74370 | 12180 | -0.6 | 99.6 | 89.8 | 5.7 |
|  |  |  | EF3 | 630600 | 97530 | 0.2 | 100 | 94.1 | 1.8 |
|  |  |  | EF4 | 485400 | 78860 | -0.4 | 100 | 93.6 | 1.7 |
|  |  |  | EF5 | 308100 | 46910 | -0.6 | 100 | 88.9 | 4.3 |
|  |  |  | HW1 | 353300 | 55280 | 0.1 | 100 | 96.3 | 1.1 |
|  |  |  | HW2 | 442500 | 70220 | -0.2 | 100 | 95.7 | 1.2 |
|  |  |  | INF1 | 393300 | 62520 | -0.9 | 99.7 | 92.6 | 1.9 |
| **Telmisartan** | 8.72 | 515.244 | EF1 | 764900 | 132000 | 0.1 | 100 | 97.0 | 1 |
|  |  |  | EF2 | 682300 | 122400 | -0.1 | 100 | 96.4 | 1.2 |
|  |  |  | EF3 | 653800 | 112700 | -0.9 | 100 | 92.2 | 2.5 |
|  |  |  | EF4 | 369500 | 57430 | -1.1 | 100 | 90.1 | 3 |
|  |  |  | HW1 | 345700 | 61010 | 0.2 | 100 | 75.7 | 17.3 |
|  |  |  | HW2 | 52430 | 7962 | -0.6 | 96 | 71.1 | 21.2 |
|  |  |  | INF1 | 271200 | 44530 | -0.8 | 100 | 94.5 | 1.2 |
| **Temazepam** | 7.85 | 301.074 | EF1 | 22220 | 4025 | -0.9 | 100 | 86.1 | 7.6 |
|  |  |  | EF5 | 74280 | 13610 | -7.5 | 98.8 | 59.3 | 3 |
| **Testosterone** | 8.06 | 289.216 | INF1 | 30830 | 6295 | 3.5 | 74.3 | 53.8 | 27.8 |
| **Theobromine** | 3.67 | 181.072 | EF4 | 75490 | 8922 | -0.6 | 56.9 | 60.9 | 22.6 |
|  |  |  | EF5 | 487200 | 67100 | -5.4 | 96.6 | 68.7 | 3.8 |
|  |  |  | HW1 | 2140000 | 272200 | -1.5 | 96 | 88.2 | 1.4 |
|  |  |  | HW2 | 1482000 | 179200 | -2.2 | 96 | 87.1 | 0.7 |
|  |  |  | INF1 | 2420000 | 296000 | -1.3 | 97.9 | 91.8 | 0.7 |
| **Thymopentin** | 6.78 | 680.373 | EF2 | 369300 | 55910 | -4.9 | 100 | 65.9 | 8 |
|  |  |  | EF4 | 15940 | 3212 | -2.3 | 98.2 | 63.6 | 24.4 |
|  |  |  | HW1 | 1283000 | 198800 | -5.6 | 100 | 68.0 | 4.1 |
|  |  |  | HW2 | 1822000 | 268100 | -3.2 | 100 | 77.6 | 5.1 |
|  |  |  | INF1 | 384200 | 56220 | -5.9 | 100 | 69.9 | 1.2 |
| **Tridemorph** | 8.94 | 298.31 | EF1 | 4782 | 1481 | -0.4 | 53.8 | 60.8 | 23.7 |
|  |  |  | EF2 | 6901 | 1783 | 0 | 53.8 | 61.6 | 21.4 |
|  |  |  | EF3 | 7128 | 1729 | -3 | 53.8 | 52.5 | 16.4 |
|  |  |  | EF4 | 12910 | 2697 | 0.4 | 87.2 | 67.1 | 21.4 |
|  |  |  | HW2 | 8411 | 1902 | -1.3 | 97.8 | 63.6 | 21.7 |
|  |  |  | RW | 22510 | 5883 | 0 | 53.8 | 61.3 | 21.4 |
| **Triphenylphosphate** | 8.43 | 327.078 | EF1 | 107500 | 21520 | -0.8 | 61.1 | 81.4 | 3.5 |
|  |  |  | EF3 | 63700 | 10660 | -0.4 | 67.9 | 87.8 | 1.5 |
|  |  |  | EF4 | 79980 | 15020 | -0.6 | 88.3 | 89.7 | 3.3 |
|  |  |  | EF5 | 344000 | 70520 | -6.8 | 89.3 | 60.6 | 1.9 |
|  |  |  | HW2 | 36040 | 7309 | -1 | 55.6 | 74.8 | 6.1 |
|  |  |  | RW | 416800 | 78600 | -1.4 | 91.2 | 88.9 | 1.7 |
| **Valsartan** | 7.7 | 436.234 | EF1 | 1663000 | 286700 | 2.1 | 99.6 | 88.9 | 0.5 |
|  |  |  | EF2 | 464300 | 77830 | 0.4 | 99 | 93.5 | 2.9 |
|  |  |  | EF3 | 608800 | 100800 | -0.2 | 99.7 | 95.3 | 2 |
|  |  |  | EF4 | 1232000 | 220200 | -0.2 | 99.3 | 97.1 | 0.1 |
|  |  |  | HW1 | 1462000 | 239300 | -1.8 | 99.7 | 89.3 | 0.1 |
|  |  |  | HW2 | 720600 | 132700 | -0.8 | 99.3 | 88.4 | 4.5 |
|  |  |  | INF1 | 1100000 | 178600 | -0.3 | 99.6 | 94.4 | 2 |
| **Verapamil** | 7.01 | 455.29 | HW1 | 471200 | 77020 | -1 | 100 | 56.8 | 12.5 |
| **Verrucarol** | 6.19 | 267.159 | EF1 | 51200 | 6102 | -0.2 | 97.7 | 73.4 | 39.2 |
| **Viquidil** | 6.17 | 325.191 | EF1 | 122500 | 16370 | 3.2 | 69.3 | 66.4 | 9.3 |
